# Food preferences and mortality risk in the prospective cohort of UK Biobank participants

**DOI:** 10.1038/s41598-026-48494-3

**Published:** 2026-04-17

**Authors:** Gerrit Eichner, Mathias Fasshauer, Sylva Mareike Schaefer

**Affiliations:** 1 Mathematical Institute, Justus-Liebig University of Giessen, 35392 Giessen, Germany; 2 Institute of Nutritional Science, Justus-Liebig University of Giessen, 35390 Giessen, Germany; 3 Center for Sustainable Food Systems, Justus-Liebig University of Giessen, 35390 Giessen, Germany

**Keywords:** Food preferences, Food frequency questionnaire, Mortality, UK Biobank, Epidemiology, Nutrition

## Abstract

Food preferences may serve as less biased proxies for self-reported food intake in predicting health outcomes but have not yet been linked to mortality risk. We included 177,148 UK Biobank participants who completed a food preference questionnaire (FPQ), with a mean follow-up of 3.4 years (607,779 person-years; 3355 deaths). High versus low preference for 140 food items was tested using Cox regression with Holm adjustment. Sensitivity analyses excluded participants with key health conditions, additional supplement use, or short follow-up, added BMI and physical activity as covariates, and redefined preference groups. Higher preference for asparagus (hazard ratio (95% confidence interval)): 0.69 (0.62, 0.77)), aubergine (0.79 (0.72, 0.87)), black pepper (0.72 (0.64, 0.81)), broccoli (0.69 (0.61, 0.79)), butternut squash (0.75 (0.68, 0.83)), and extra virgin olive oil (0.67 (0.58, 0.78)) was linked to lower all-cause mortality, whereas higher preference for regular fizzy drinks (1.38 (1.24, 1.54)) was linked to higher mortality across all analyses. Specific food preferences are associated with all-cause mortality risk. FPQs may, therefore, complement conventional dietary assessments. Further studies should examine their associations with metabolic and cardiovascular outcomes.

**Supplementary Information:**

The online version contains supplementary material available at 10.1038/s41598-026-48494-3.

## Introduction

Food consumption significantly impacts health and contributes to mortality globally^1^. Food preferences are widely recognized as central indicators of dietary behaviour, playing a significant role in shaping eating habits and serving as a potent predictor for energy consumption and quantity consumed during a meal^2,3^. Without economic and availability constraints, food preferences might be the single most important determinant of food choice in meals^4–6^. If foods are not perceived positively in their appearance, smell, texture, or taste, they are unlikely to be eaten^7^.

Nowadays, food frequency questionnaires (FFQs) are the most commonly used tools in nutritional epidemiology research due to their cost-effectiveness, simplicity, and capacity to capture dietary behaviour at different time points^8^. However, FFQs face challenges when measuring food choices since responses to these questionnaires are often subject to biases, such as, e.g., the social desirability bias^9^. In addition, memory and health status can influence the perception of participants and, thus, reported intakes^10,11^. Furthermore, high dietary restraint, occurring especially in obese participants^12^, may be associated with a reduction in reporting of consumed foods, limiting the accuracy of dietary reporting in FFQs^13^. The use of FFQs among low-literacy, low-income, and minority respondents often poorly captures dietary habits^14,15^.

Food preference questionnaires (FPQs), on the other hand, can serve as valuable tools for understanding dietary behaviour by capturing food choices based on likes and dislikes. In contrast to factual-memory-based FFQs, FPQs rely on affective memory which can be assessed even when factual memory might be compromised^16,17^. Moreover, food preferences are stable over time in adult populations and FPQs have shown good test–retest reliability^18^ compared to FFQs^19^. Different studies have reported advantages of using FPQs in comparison to FFQs to assess health outcomes^17,20,21^. Thus, a positive correlation between preference for sweet foods and caries prevalence was found, while no such association was observed with reported simple sugar intake^20^. Moreover, food preferences for fat and fibre outperformed reported fat and fibre consumption in explaining variations in adiposity and blood pressure^17,21^. In a recent Nutrinet-Santé cohort study, liking for fat was used alongside consumption measures as a determinant of dietary fat intake^22^.

Previous research on food preferences has faced challenges such as relatively small sample sizes, rather homogeneous populations, or only one sex being studied^2,23–26^. Furthermore, while different food groups have convincingly been associated with higher or lower risk of all-cause mortality^27^, no such data exists for food preferences. Therefore, the current study investigates for the first time the association between self-reported food preferences and all-cause mortality in 177,148 participants of the UK Biobank study. We argue that FPQ data offers a novel approach to capturing dietary behaviour and its impact on health outcomes.

## Methods

### Study design

The UK Biobank study is a large-scale prospective cohort study, initiated between 2006 and 2010. It comprises a cohort of over 500,000 individuals aged between 40 and 69 years who were recruited from 22 assessment centres across the UK^28,29^. At baseline, all participants provided written informed consent, and ethical approval for the study was issued by the North West Multicentre Research Ethics Committee^28,29^. Participants’ health outcomes were derived from self-reported data collected during UK Biobank assessment centre visits, as well as from linked electronic health data, including hospital inpatient records and primary care data^28,29^.

### Assessment of food preferences

Food-preference phenotypes were assessed using an online questionnaire containing 150 items, i.e., various foods, drinks, and additional non-food items related to health behaviours such as smoking or physical activity. The FPQ includes a variety of elements which have been previously used in population-based cohorts around the world, e.g. the Italian taste projects, the Erasmus Rucphen Family Study, Twins UK, and the Viking Health Study^11^. Before inviting all UK Biobank participants with a contact email address to complete the questionnaire, it was piloted with 10,000 participants to ensure adequacy of content and length^11^. The FPQ was designed to harmonise international research on food preferences. A core set of items commonly found worldwide was identified through consultation with scientists from the USA, UK, Italy, the Netherlands, and Australia. The questionnaire was subsequently refined to reflect the UK diet and terminology. It aims to cover most food groups while remaining simple and acceptable in length for respondents^11^. The FPQ’s food items were designed to reflect both sensory preferences (e.g., bitter, sweet) and preferences for specific food categories (e.g., fruits, vegetables, meat). At least five items for each preference category were included in the questionnaire. In 2019, this questionnaire was sent to all UK Biobank participants who had agreed to be contacted again as part of the study. The questionnaire has been designed to include a wide range of food categories while maintaining simplicity and ensuring that its length remained acceptable to respondents^11^. Due to its online administration, visual aids such as pictures were omitted, and responses were recorded as ratings using a 9-point Hedonic scale, ranging from “Extremely dislike” (1) to “Extremely like” (9). Participants also had the option to indicate if they had never tried a particular item or to choose not to answer, leading to a missing value for that food item. Furthermore, the order of the questionnaire items was randomised on a participant basis to reduce potential bias due to tiredness^11^. The complete questionnaire is available at https://biobank.ndph.ox.ac.uk/showcase/showcase/docs/foodpref.pdf.

Although relatively few studies have directly compared FPQs with FFQs, available evidence suggests that food preferences are associated with reported habitual intake. Several studies in both adolescents and adults have reported moderate to strong correlations between stated liking and frequency of consumption for corresponding food items or food groups. For example, Andreatta et al. observed significant correlations between preference ratings and consumption frequency assessed by FFQ in an adult Argentine population (Spearman’s ρ ≈ 0.50)^30^. Similarly, studies in adolescents have demonstrated substantial associations between preference scores and reported intake frequencies, with correlation coefficients for specific food groups reaching up to approximately 0.80 in some cases^31^. More recently, Nagai et al. reported significant correlations between nutrient-specific preference scores and dietary intake assessed by FFQ in a Japanese cohort^32^. May-Wilson et al. demonstrate that food preferences assessed via the FPQ strongly correspond to actual consumption measured via FFQ, reporting very strong genetic correlations between corresponding traits (r > 0.7)^33^. Collectively, these findings suggest that while preference and intake are not interchangeable constructs, they show consistent and meaningful associations across populations, supporting the biological and behavioural link between liking and habitual consumption.

### Participant selection and exclusion criteria

Figure S1 illustrates the participant selection. Initially, participants from the UK Biobank pilot phase were removed from the analysis (n = 3,794; Fig. S1) due to differences in questionnaire items compared to the later cohort. Subsequently, individuals who did not complete the FPQ were excluded (n = 317,514). Additionally, 10 of the 150 questionnaire items were excluded because they pertained to preferences unrelated to food such as smoking and physical activity (Table S1) similar to the methodology applied by Navratilova and colleagues^34^. All food preference items included in the analysis are listed in Table S2. Lastly, the following exclusion criteria were applied: (1) missing smoking status, (2) missing socioeconomic factors (ethnic background, highest qualification, overall health rating, total household income or Townsend index), (3) missing data from the physical exam (body mass index (BMI), systolic blood pressure), and (4) implausible follow-up time (Figs. S1 and S2). Thus, a total of 177,148 participants and 140 food preference items could be included in the study.

### Outcome assessment

Mortality data of UK Biobank participants was acquired from NHS England for individuals in England and Wales, and from the NHS Central Register for participants from Scotland^35^. Further details about the linkage to death registries can be found under https://biobank.ndph.ox.ac.uk/showcase/showcase/docs/DeathLinkage.pdf. Follow-up time was defined as the duration between filling out the FPQ and date of death, loss to follow-up, or end of data acquisition (December 2022), whichever came first and where the latter two led to censored observations.

### Statistical analyses

Data analysis was performed with R version 4.5.0^36^ and, among others, the R packages survival^37^ and forestploter^38^. The 9-point hedonic scale was divided into three groups according to the preference ratings that the participants indicated for each item, i.e., low preference: 1–3, medium preference: 4–6, and high preference: 7–9. Hazard ratios (HRs) with their pointwise 95% confidence intervals (CI) for all-cause mortality were assessed in Cox proportional hazard regression models comparing the high preference to the low preference group for each preference item. A directed acyclic graph (DAG) showing hypothesized causal relationships that underlie the association between food preferences and all-cause mortality was used to identify an appropriate set of confounding variables using the R package DAGitty^39,40^ to assess an unconfounded effect estimate. Hence, the Cox models were adjusted for the following covariates as summarized in Fig. S3: Age at FPQ assessment (split by quintiles), ethnic background (White, Non-White (Mixed, Asian, Black, and other)), general health status (poor, fair, good, excellent), highest qualification (none of the below, national exams at age 16 years, vocational qualifications or optional national exams at ages 17–18 years, professional, College or University), sex (female, male), and smoking status (never, previous, current occasional, current < 10, 10 to 14, 15 to 19, ≥ 20 cigarettes per day). Since the proportional hazards assumption was not significantly violated, as assessed using the cox.zph function based on scaled Schoenfeld residuals, stratification of the respective covariates was not required in the final models. If the high preference group was statistically significantly different from the low preference group with a *p*-value < 0.05 after employing Holm’s adjustment method for multiple testing across the family of all 140 items, it was included in the final presentation of results. Holm’s method was applied to control the family-wise error rate at 0.05, providing a balanced and conservative correction that limits false positives while being less stringent than Bonferroni and more restrictive than false discovery rate methods. Furthermore, the unadjusted *p*-values for the respective pointwise HR are given for all significant preference items. The number of participants included in the respective analysis of each food preference item is illustrated in Table S2. All food preferences with a Holm-adjusted *p*-value ≥ 0.05 are listed in Fig. S4. All exact *p*-values of the main and sensitivity analyses can be found in Table S3.

The following sensitivity analyses were performed to evaluate the robustness of the results: Firstly, participants who reported unintentional weight loss were removed from the study as this might be a sign of, e.g., malignant diseases, frailty, or psychological disorders (n = 28,065)^41^. To address reverse causation, participants who died or were lost to follow-up within the first year after FPQ completion (landmark analysis) were excluded from the study cohort (n = 708). Furthermore, models were additionally adjusted for BMI or MET (metabolic equivalent of task)-minutes per week to assess if the effect of the food preferences was mediated through body weight or physical activity. Moreover, the low and the high preference groups were extended to 1–4 as the low preference group and 6–9 as the high preference group to assess whether significances of food preference items remain robust even when the low or high preference is not very strong. To address potential bias from medical conditions that may affect food choices through dietary restrictions, several sensitivity analyses were performed excluding participants with baseline reports of diabetes mellitus (n = 5804), impaired kidney function (glomerular filtration rate (GFR) < 60 mL/min/1.73 m^2^) (n = 10,546), psychiatric disorders (n = 11,368), cancer (n = 13,524), or cardiovascular disease (n = 5973). To evaluate the potential influence of dietary supplement use on nutrient intake and related health outcomes, a sensitivity analysis was conducted excluding participants who reported the use of vitamin or mineral supplements (n = 58,208). Furthermore, analyses were stratified by sex and smoking status (non-smokers, previous smokers, and current smokers).

## Results

### Baseline data of UK Biobank participants

In total, 177,148 participants were included in the present study (Fig. S1). The baseline characteristics of the population after applying the exclusion criteria are presented in Table 1. The mean (standard deviation (SD)) age of the study cohort at FPQ completion was 66 (8) years and 57.3% of the participants were female. The follow-up period was 3.4 (0.3) years, i.e., 607,779 person-years in which 3355 deaths occurred. Demographic participant characteristics by food groups as identified by Concas et al.^23^ are presented in Tables S4 and S5. Baseline characteristics of UK Biobank participants who completed and did not complete the FPQ are shown in Table S6.

**Table 1:** Baseline characteristics of the UK Biobank cohort.

Parameters	Total cohort (n = 177,148)
Age at completion of FPQ	66 (8)
Sex—female	101,563 (57.3)
BMI (kg/m^2^)	
Underweight (< 18.5 kg/m^2^)	992 (0.6)
Normal (18.5–25 kg/m^2^)	68,384 (38.6)
Overweight (25–30 kg/m^2^)	73,209 (41.3)
Obese (> 30 kg/m^2^)	34,563 (19.5)
Ethnic background	
White	172,121 (97.2)
Mixed, Asian, Black, Chinese, and other	5,027 (2.8)
General health status	
Poor	4,050 (2.3)
Fair	27,476 (15.5)
Good	106,981 (60.4)
Excellent	38,641 (21.8)
Highest qualification	
None of the below	12,553 (7.1)
National exams at age 16 years	25,390 (14.3)
Vocational qualifications or optional national exams at ages 17–18 years	30,986 (17.5)
Professional	28,135 (15.9)
College or University	80,084 (45.2)
Smoking status	
Never	103,214 (58.3)
Previous	62,138 (35.1)
Occasional	4,217 (2.4)
Current < 10 cigarettes per day	2,071 (1.2)
Current 10 to 14 cigarettes per day	1,769 (1.0)
Current 15 to 19 cigarettes per day	1,471 (0.8)
Current ≥ 20 cigarettes per day	2,268 (1.3)
Follow-up period (years)	3.4 (0.3)

Categorical variables are summarised as frequencies (percentages) and continuous variables as mean (standard deviation).

BMI, body mass index; FPQ, food preference questionnaire.

### Main analysis

**Fig. 1:**
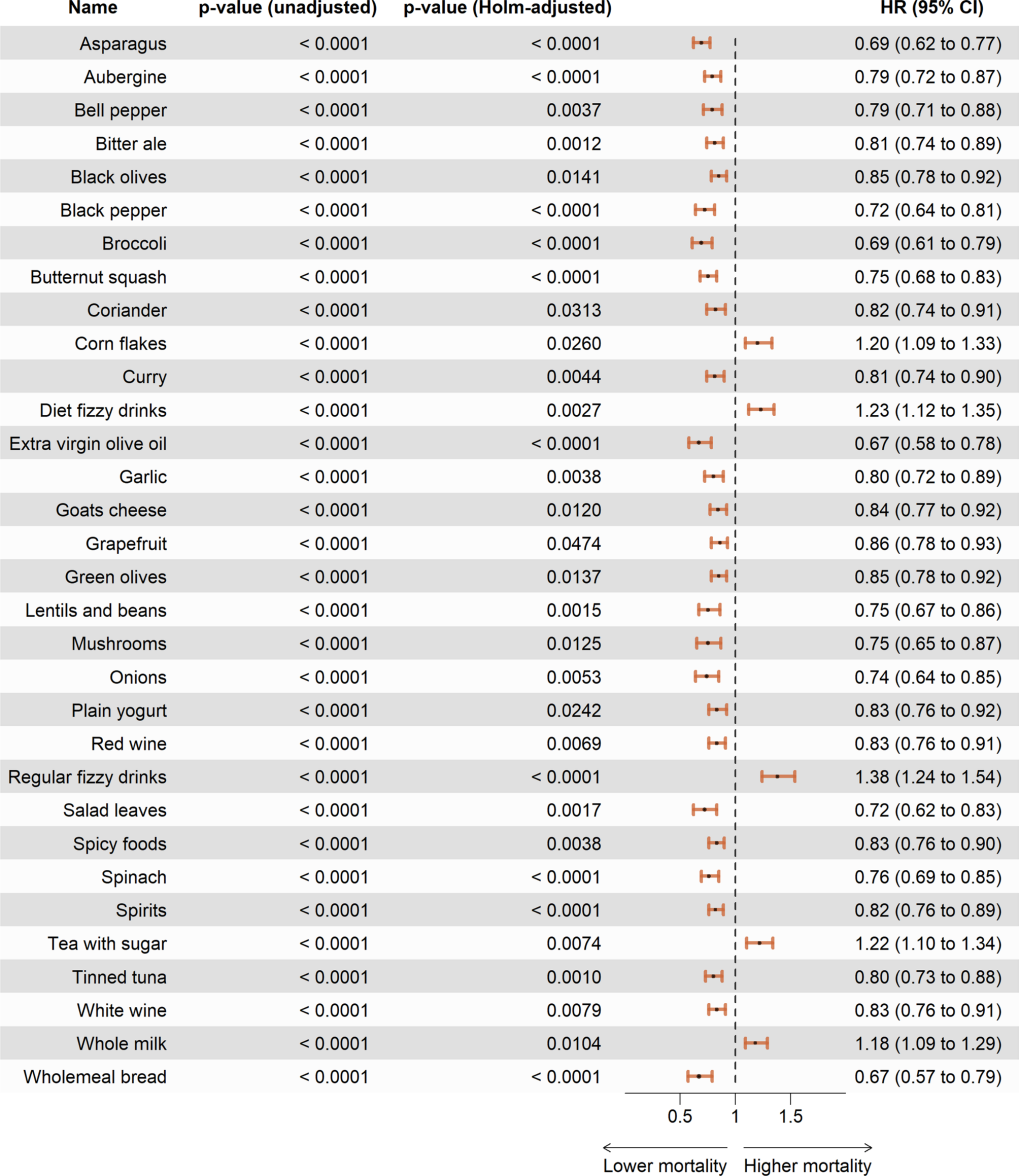
Food preferences significantly associated with all-cause mortality. Associations between high preference for a food preference item compared to low preference and all-cause mortality. Food preference items are included if their Holm-adjusted *p*-value is < 0.05. Besides the Holm-adjusted *p*-value, the unadjusted *p*-value is included with its corresponding HR (95% CI). Models are further adjusted for the following covariates: Age at FPQ assessment (split by quintiles), ethnic background (White, Non-white (Mixed, Asian, Black, and other)), general health status (poor, fair, good, excellent), highest qualification (none of the below, national exams at age 16 years, vocational qualifications or optional national exams at ages 17–18 years, professional, College or University), sex (female, male), and smoking status (never, previous, current occasional, current < 10, 10 to 14, 15 to 19, ≥ 20 cigarettes per day). Abbreviations: FPQ, Food preference questionnaire; HR, Hazard Ratio.

Figure 1 depicts all food preference items showing significant differences in all-cause mortality when comparing the high preference to the low preference group. A significantly higher HR (95% CI) for all-cause mortality was found for high preference of the following items: corn flakes (1.20 (1.09, 1.33)), diet fizzy drinks (1.23 (1.12, 1.35)), regular fizzy drinks (1.36 (1.24, 1.54)), tea with sugar (1.22 (1.10, 1.34)), and whole milk (1.18 (1.09, 1.29)). A significantly lower HR (95% CI) for all-cause mortality was found for high preference of the following 27 items: asparagus (0.69 (0.62, 0.77)), aubergine (0.79 (0.72, 0.87)), bell pepper (0.79 (0.71, 0.88)), bitter ale (0.81 (0.74, 0.89)), black olives (0.85 (0.78, 0.92)), black pepper (0.72 (0.64, 0.81)), broccoli (0.69 (0.61, 0.79)), butternut squash (0.75 (0.68, 0.83)), coriander (0.82 (0.74, 0.91)), curry (0.81 (0.74, 0.90)), extra virgin olive oil (0.67 (0.58, 0.78)), garlic (0.80 (0.72, 0.89)), goats’ cheese (0.84 (0.77, 0.92)), grapefruit (0.86 (0.78, 0.93)), green olives (0.85 (0.78, 0.92)), lentils and beans (0.75 (0.67, 0.86)), mushrooms (0.75 (0.65, 0.87)), onions (0.74 (0.64, 0.85)), plain yogurt (0.83 (0.76, 0.92)), red wine (0.83 (0.76, 0.91)), salad leaves (0.72 (0.62, 0.83)), spicy foods (0.83 (0.76, 0.89)), spinach (0.76 (0.69, 0.85)), spirits (0.82 (0.76, 0.89)), tinned tuna (0.80 (0.73, 0.88)), white wine (0.83 (0.76, 0.91)), and wholemeal bread (0.67 (0.57, 0.79)).

### Sensitivity analyses

Sensitivity analyses comprised exclusion of participants with unintentional weight loss (Fig. S5), follow-up < 1 year (Fig. S6), diabetes mellitus (Fig. S7), GFR < 60 mL/min/1.73 m^2^ (Fig. S8), psychiatric disease (Fig. S9), cancer (Fig. S10), cardiovascular disease (Fig. S11), or vitamin/mineral supplement use (Fig. S12); inclusion of BMI (Fig. S13) and physical activity (Fig. S14) as covariates; and redefinition of low and high preference groups (Fig. S15). A higher preference for regular fizzy drinks remained significantly associated with higher all-cause mortality risk across all 11 sensitivity analyses (Figs. S5–S15). Conversely, higher preferences for asparagus, aubergine, black pepper, broccoli, butternut squash, and extra virgin olive oil remained significantly associated with lower all-cause mortality (Figs. S5–S15). Preference for bitter ale, spicy foods, spinach, spirits, tea with sugar, and wholemeal bread remained significant in 10 out of 11 sensitivity analyses, while preferences for curry, lentils and beans, and tinned tuna remained significant in 9 out of 11 sensitivity analyses. Other food preference items that were significant in the main analysis were not consistently significant throughout all the sensitivity analyses (Figs. S5–S15).

### Stratified analyses

When analyses were stratified by sex, several associations observed in the main analysis remained significant (Figs. S16 and S17). Among females, higher preference for asparagus, beetroot, black pepper, and butternut squash was associated with lower mortality risk, whereas preferences for regular fizzy drinks and whole milk were associated with higher mortality risk.

Among males, higher preference for asparagus, bitter ale, broccoli, butternut squash, extra virgin olive oil, lentils and beans, spinach, spirits, tinned tuna, and wholemeal bread was associated with lower mortality risk, while higher preference for diet and regular fizzy drinks was associated with higher mortality risk (Figs. S16 and S17). When stratified by smoking status, several associations remained significant among non-smokers, including preferences for asparagus, black pepper, butternut squash, extra virgin olive oil, spinach, and regular fizzy drinks (Fig. S18). Full results of the stratified analyses are presented in the supplementary figures.

## Discussion

### Principal findings

This is the first study linking food preferences with all-cause mortality. Of the 140 studied food items, a high preference for the following items is linked with lower mortality risk across all analyses: asparagus, aubergine, black pepper, broccoli, butternut squash, extra virgin olive oil. In contrast, a high preference for regular fizzy drinks is associated with higher mortality risk.

### Comparison with preference studies

No previous prospective studies have examined how food preferences relate to all-cause mortality or morbidity. Most existing work is cross-sectional or experimental, linking preferences to dietary intake, obesity, and cardiometabolic risk factors^21,33,34^. These studies indicate that food preferences mirror dietary behaviours relevant to long-term health. The present analysis extends this evidence by showing that specific preferences predict mortality risk.

A cross-sectional study of 422 men finds that low preference for fibre-rich foods is associated with higher BMI and waist circumference which are both predictors of elevated blood pressure and adverse lipid profiles^21^. This aligns with our observation that preference for fibre-rich vegetables such as asparagus, aubergine, broccoli, and butternut squash corresponds to lower mortality. In a larger cross-sectional analysis of 161,625 UK Biobank participants, higher liking for highly palatable foods is genetically correlated with higher BMI, greater body-fat percentage, and lower socioeconomic status^33^. This pattern matches our observation that strong preference for items such as regular fizzy drinks and sweetened tea is associated with higher mortality risk. Navratilova and colleagues^34^ identify three preference clusters using machine learning: *Health-conscious* (high preference for vegetables and fruit, low for animal-based and sweet foods), *Omnivore* (high preference across foods), and *Sweet-tooth* (high preference for sweet foods and drinks). Participants in the Health-conscious group show lower risk of chronic disease than those in the other two groups^34^. This aligns with our finding that higher preference for vegetables and fibre-rich foods and lower preference for sugar-sweetened drinks correspond to lower mortality risk.

### Comparison with FFQ studies

While food preference and food intake assess distinct dimensions of dietary behaviour^17^, both reflect underlying dietary patterns that are closely linked to health outcomes. FFQs capture habitual intake over a defined period, whereas FPQs measure individual liking and aversion, which might, even though they do not directly quantify, influence and determine consumption. Against this background, it is relevant to examine whether the associations observed in the present study align with findings from FFQ-based research. Higher preference for vegetables such as asparagus, aubergine, broccoli, butternut squash, and spinach is consistently linked to lower mortality. This aligns with large FFQ-based meta-analyses showing that greater vegetable and fibre consumption reduces all-cause mortality across populations of more than 1.5 million participants^42,43^. These relationships likely reflect dietary habits rich in nutrient-dense, high-fibre foods that lower e.g. cardiometabolic risk through improved lipid metabolism, enhanced insulin sensitivity, reduced inflammation, and favourable gut microbiota modulation^44,45^. Moreover, specific items such as black pepper^46^ and extra virgin olive oil^47^ have been linked to lower mortality risk, consistent with our FPQ findings. Oleuropein and hydroxytyrosol from olive sources may improve cardiometabolic health by enhancing insulin sensitivity through AMP-activated protein kinase GLUT4 signalling and reducing oxidative stress, with studies showing improvements in glucose metabolism, lipid profiles, and inflammatory markers^48,49^. Black pepper, through its bioactive compound piperine, may protect against cardiovascular and inflammatory diseases by inhibiting pro-inflammatory cytokines, reducing oxidative stress, and promoting healthy lipid metabolism and endothelial function^50,51^.

Conversely, stronger preferences for sugar-sweetened beverages (SSB), including regular fizzy drinks and sweetened tea, are associated with higher mortality in the main analysis, with regular fizzy drinks remaining significant across all sensitivity analyses. Large meta-analyses of 11 cohorts including 965,851 participants and of 40 prospective studies including 5,750,133 participants demonstrate that sugar-and artificially-sweetened beverages are linked to higher mortality risk^52,53^. In contrast, no significant association between added sugar in coffee or tea and mortality is found in the Copenhagen Male Study^54^ or in our own previous work^55^. Inconsistencies across studies using FFQs may stem from differences in questionnaire design, recall periods, and food item lists, which affect how dietary intake is captured. Variation in portion size estimation, cultural eating patterns, and reporting accuracy can further contribute to heterogeneity in results across populations and study settings. Nevertheless, a preference for sweet beverages, especially SSB, may serve as a behavioural marker for higher free sugar intake, which can elevate mortality risk as shown in several studies^52,53,55–57^. This could be conveyed via various pathways and mechanisms such as increased obesity, insulin resistance, dyslipidaemia, and chronic low-grade inflammation^58^. Sensitivity analyses extending low and high preference groups further suggest that even modest preferences influence mortality risk, as exemplified by asparagus, where a slight positive preference (≥ 6) still predicts lower mortality compared to a slight negative preference (≤ 4).

Our study suggests that food preferences can serve as a practical indicator of habitual dietary patterns linked to mortality risk. Identifying preference profiles associated with higher risk may help target groups for dietary intervention. The FPQ’s simplicity makes it suitable for large-scale population studies and digital health applications where detailed dietary assessment is not feasible. Future research should examine how preferences relate to metabolic and cardiovascular disease and validate FPQ findings using objective intake data and biomarkers.

In additional stratified analyses by sex and smoking status, several of the observed associations remained significant, particularly among non-smokers. These findings suggest that the associations between food preferences and mortality risk are largely consistent across population subgroups, although some differences may exist depending on lifestyle factors such as smoking. However, these subgroup analyses should be interpreted cautiously, as the subgroup sizes differ substantially, particularly for smoking status, and require confirmation in future studies.

### Strengths and limitations

The strengths of the current study encompass the large-scale, prospective cohort design, a detailed participant characterisation, a wide range of food preference items included in the questionnaire, and thorough adjustment for multiple testing via Holm adjustment. Some limitations of our findings must be acknowledged. Due to the observational nature of the study, causality cannot be derived. The FPQ used in this analysis has not been formally validated, which may affect the reliability of the dietary assessment. The self-reported nature of food preferences introduces potential misclassification bias, which could attenuate or distort the observed associations. Nonetheless, recall bias is likely limited, as the FPQ assesses individual preferences through affective-memory-based questions rather than requiring respondents to recall their past dietary intake, as outlined by Jilani and colleagues^59^. In addition, food preference ratings do not necessarily correspond directly to actual consumption patterns, as intake may be influenced by factors such as perceived healthiness, cost, or availability. Moreover, the questionnaire captures hedonic preference rather than frequency or quantity of intake. The FPQ also includes a limited range of food items and therefore may not fully capture the variability of participants’ overall dietary patterns (e.g., meat alternatives), which may have led some participants to select items they perceived as similar rather than indicating non-consumption. Furthermore, UK Biobank participants tend to be healthier, more educated, and older compared with the general population, leading to possible selection bias^60^. Table S6 indicates that participants who completed the FPQ were more likely to be female, had a lower BMI, and reported better overall health status. They were also more highly educated, more likely to be of White ethnic background, and less likely to be current smokers. In contrast, those who did not complete the FPQ had higher proportions of obesity, poorer health, and lower educational attainment. A further limitation of this study is that energy intake was not assessed within the food preference cohort and thus could not be adjusted for. Lastly, external validity may be limited due to the mostly West-European sample, requiring replication in studies involving non-Western European populations.

## Conclusions

In this study, certain food preferences including various vegetables, fibre-rich starchy foods, and extra virgin olive oil are associated with a lower mortality risk, whereas a preference for SSB is linked with a higher risk of death. Therefore, FPQs might add valuable information to dietary assessment and may help predict all-cause mortality risk. Further studies should validate the FPQ findings using objective biomarkers and assess associations with metabolic and cardiovascular disease.

## Supplementary Information

Supplementary Information.

## Data Availability

The data that support the findings of this study are available from UK Biobank but restrictions apply to the availability of these data, which were used under license for Application 53,438, and, therefore, are not publicly available. Bona fide researchers can apply to use the UK Biobank dataset by registering and applying at [https://www.ukbiobank.ac.uk/enable-your-research/register].

## Abbreviations

BMI: Body mass index
CI: Confidence interval
DAG: Directed acyclic graph
FFQ: Food frequency questionnaire
FPQ: Food preference questionnaire
HR: Hazard ratio
MET: Metabolic equivalent of task
SSB: Sugar-sweetened beverages
SD: Standard deviation
