# Supplementary material for: Food preferences and mortality risk in the prospective cohort of UK Biobank participants
Source: Sci Rep. 2026 Apr 17;16:12727. doi: 10.1038/s41598-026-48494-3 (PMC13090334; doi:10.1038/s41598-026-48494-3)
Supplement: Supplementary file 1 — Supplementary Information. [file 41598_2026_48494_MOESM1_ESM.pdf]

Supplementary Material

Scientific Reports

Association of food preferences and risk of mortality in the prospective cohort of UK Biobank participants

Gerrit Eichner, PhD[1]; Mathias Fasshauer, M.D.[2,3#]; Sylva Mareike Schaefer, PhD[2#]

1. Mathematical Institute, Justus-Liebig University of Giessen, Giessen, Germany.
2. Institute of Nutritional Science, Justus-Liebig University of Giessen, Giessen, Germany.
3. Center for Sustainable Food Systems, Justus-Liebig University of Giessen, Giessen, Germany.

[#]SMS and MF contributed equally to this work and are joint senior authors.

Corresponding author

Sylva Mareike Schaefer

Institute of Nutritional Science

Justus-Liebig University of Giessen, 35390 Giessen, Germany

Phone: +49 641 9939067; E-mail: sylva.schaefer@uni-giessen.de

ORCID: 0000-0001-5949-3282

Index

Supplementary Figures

Figure S1

Flowchart of participant selection

Abbreviations: FPQ, Food preference questionnaire

Figure S2

Venn Diagram of exclusion criteria

The following exclusion criteria were applied to all analyses: 1) missing smoking status, 2) missing socioeconomic factors (Townsend deprivation index, total household income, ethnic background, highest qualification, or overall health rating), 3) missing data of the physical exam (body mass index (BMI), systolic blood pressure (SBP)), 4) implausible follow-up time.

Figure S3

Directed acyclic graph for covariate selection

Directed acyclic graph representing the assumed relationships between variables that may affect the relationship between food preferences (green disc with inscribed triangle; exposure) and all-cause mortality risk (blue disc with inscribed vertical bar; outcome). Other blue discs denote ancestors of the outcome, i.e., direct or indirect predecessors of the outcome; white discs denote covariates for which the model was adjusted, i.e., age, ethnic background, highest qualification, general health status, sex, and smoking. Dark/light grey discs denote other variables that are not influential or unobserved and pink discs denote ancestors of both exposure and outcome. Green arrows indicate causal paths. This graph is used to identify confounding variables for model adjustment and was created by using the R package “dagitty”, based on the web tool dagitty.net (1, 2). Abbreviations: BMI, Body mass index; MET, Metabolic equivalent of task

Figure S4

All food preference items with a Holm-adjusted p-value > 0.05

Associations between high preference for a food preference item compared to low preference and all-cause mortality. Besides the Holm-adjusted p-value, the unadjusted p-value is included with its corresponding pointwise HR (95% CI).

Abbreviations: CI, Confidence interval; HR, Hazard ratio

Figure S5

Participants with unintentional weight loss removed

Associations between high preference for a food preference item compared to low preference and all-cause mortality (n = 149,083). Only food preference items that were significant in the main analysis are included. Besides the Holm-adjusted p-value, the unadjusted p-value is included with its corresponding pointwise HR (95% CI). Models are assessed within a cohort where all participants reporting unintentional weight loss (n = 28,065) are removed. Models were adjusted similarly to the main analysis (Fig. 1).
Abbreviations: CI, Confidence interval; HR, Hazard ratio

Figure S6

Landmark analysis (up to 1 year follow-up excluded)
Associations between high preference for a food preference item compared to low preference and all-cause mortality (n = 176,440). Only food preference items that were significant in the main analysis are included. Besides the Holm-adjusted p-value, the unadjusted p-value is included with its corresponding pointwise HR (95% CI). Models are assessed within a cohort where all participants with a follow up time of < 1 year (n = 708) are excluded. Models were adjusted similarly to the main analysis (Fig. 1).
Abbreviations: CI, Confidence interval; HR, Hazard ratio

Figure S7

Participants with diabetes mellitus excluded
Associations between high preference for a food preference item compared to low preference and all-cause mortality (n = 171,344). Only food preference items that were significant in the main analysis are included. Besides the Holm-adjusted p-value, the unadjusted p-value is included with its corresponding pointwise HR (95% CI). Models are assessed within a cohort where all participants who indicated a diabetes mellitus diagnosis at baseline assessment (n = 5,804) were removed. Models were adjusted similarly to the main analysis (Fig. 1).
Abbreviations: CI, Confidence interval; HR, Hazard ratio

Figure S8

Participants with GFR ≥ 60 at baseline assessment
Associations between high preference for a food preference item compared to low preference and all-cause mortality (n = 166,602). Only food preference items that were significant in the main analysis are included. Besides the Holm-adjusted p-value, the unadjusted p-value is included with its corresponding pointwise HR (95% CI). Models are assessed within a cohort where all participants who indicated a GFR below 60 mL/min/1.73 m^2at baseline assessment (n = 10,546) were removed. Models were adjusted similarly to the main analysis (Fig. 1).
Abbreviations: CI, Confidence interval; HR, Hazard ratio

Figure S9

Participants without history of any psychiatric disease

Associations between high preference for a food preference item compared to low preference and all-cause mortality (n = 165,780). Only food preference items that were significant in the main analysis are included. Besides the Holm-adjusted p-value, the unadjusted p-value is included with its corresponding pointwise HR (95% CI). Models are assessed within a cohort where all participants with a history of any psychological disease at baseline assessment (n = 11,368) were removed. Models were adjusted similarly to the main analysis (Fig. 1). Abbreviations: CI, Confidence interval; HR, Hazard ratio

Figure S10

Participants with history of cancer excluded

Associations between high preference for a food preference item compared to low preference and all-cause mortality (n = 163,624). Only food preference items that were significant in the main analysis are included. Besides the Holm-adjusted p-value, the unadjusted p-value is included with its corresponding pointwise HR (95% CI). Models are assessed within a cohort where all participants with a history of cancer indicated at baseline assessment (n = 13,524) were removed. Models were adjusted similarly to the main analysis (Fig. 1). Abbreviations: CI, Confidence interval; HR, Hazard ratio

Figure S11

Participants with history of cardiovascular disease excluded

Associations between high preference for a food preference item compared to low preference and all-cause mortality (n = 171,175). Only food preference items that were significant in the main analysis are included. Besides the Holm-adjusted p-value, the unadjusted p-value is included with its corresponding pointwise HR (95% CI). Models are assessed within a cohort where all participants with a history of cardiovascular disease indicated at baseline assessment (n = 5,973) were removed. Models were adjusted similarly to the main analysis (Fig. 1). Abbreviations: CI, Confidence interval; HR, Hazard ratio

Figure S12

Participants not taking any vitamin or mineral supplements

Associations between high preference for a food preference item compared to low preference and all-cause mortality (n = 118,940). Only food preference items that were significant in the main analysis are included. Besides the Holm-adjusted p-value, the unadjusted p-value is included with its corresponding pointwise HR (95% CI). Models are assessed within a cohort

where all participants taking vitamin or mineral supplements at baseline assessment (n = 58,208) were excluded. Models were adjusted similarly to the main analysis (Fig. 1). Abbreviations: CI, Confidence interval; HR, Hazard ratio

Figure S13

Inclusion of BMI into the model

Associations between high preference for a food preference item compared to low preference and all-cause mortality (n = 177,148). Only food preference items that were significant in the main analysis are included. Besides the Holm-adjusted p-value, the unadjusted p-value is included with its corresponding pointwise HR (95% CI). Associations are assessed adding BMI to the model formula. Models were adjusted for age, BMI, general health status, highest qualification, sex, and smoking status.

Abbreviations: BMI, Body Mass Index; CI, Confidence interval; HR, Hazard ratio

Figure S14

Inclusion of physical activity (MET per week) into the model

Associations between high preference for a food preference item compared to low preference and all-cause mortality (n = 177,148). Only food preference items that were significant in the main analysis are included. Besides the Holm-adjusted p-value, the unadjusted p-value is included with its corresponding pointwise HR (95% CI). Associations are assessed adding physical activity in MET minutes per week to the model formula. Models were adjusted for age, ethnic background, general health status, highest qualification, physical activity, sex, and smoking status. Abbreviations: CI, Confidence interval; HR, Hazard ratio; MET, Metabolic equivalent of task

Figure S1

Larger groups by adding level 4 to the low preference group and 6 to the high preference group

Associations between high preference for a food preference item compared to low preference and all-cause mortality (n = 177,148). Only food preference items that were significant in the main analysis are considered. The low food preference group is extended by the value 4 and the high food preference group is extended by the value 6 compared to the main analysis. Besides the Holm-adjusted p-value, the unadjusted p-value is included with its corresponding pointwise HR (95% CI). Models were adjusted similarly to the main analysis (Fig. 1).

Abbreviations: CI, Confidence interval; HR, Hazard ratio

Figure S16

Females only

Associations between high preference for a food preference item compared to low preference and all-cause mortality in female participants only (n = 101,563). Only food preference items that were significant in the main analysis are included. Besides the Holm-adjusted p-value, the unadjusted p-value is included with its corresponding pointwise HR (95% CI). Models were adjusted for age, ethnic background, general health status, highest qualification, physical activity, and smoking status. Abbreviations: CI, Confidence interval; HR, Hazard ratio

Figure S17

Males only

Associations between high preference for a food preference item compared to low preference and all-cause mortality in male participants only (n = 75,585). Only food preference items that were significant in the main analysis are included. Besides the Holm-adjusted p-value, the unadjusted p-value is included with its corresponding pointwise HR (95% CI). Models were adjusted for age, ethnic background, general health status, highest qualification, physical activity, and smoking status. Abbreviations: CI, Confidence interval; HR, Hazard ratio

Figure S18

Non-smokers only.

Associations between high preference for a food preference item compared to low preference and all-cause mortality in non-smoking participants only (n = 103,214). Only food preference items that were significant in the main analysis are included. Besides the Holm-adjusted p-value, the unadjusted p-value is included with its corresponding pointwise HR (95% CI). Models were adjusted for age, ethnic background, general health status, highest qualification, physical activity, and sex. Abbreviations: CI, Confidence interval; HR, Hazard ratio

Figure S19

Previous smokers only.

Associations between high preference for a food preference item compared to low preference and all-cause mortality in previous smokers only (n = 62,138). Only food preference items that were significant in the main analysis are included. Besides the Holm-adjusted p-value, the unadjusted p-value is included with its corresponding pointwise HR (95% CI). Models were adjusted for age, ethnic background, general health status, highest qualification, physical activity, and sex. Abbreviations: CI, Confidence interval; HR, Hazard ratio

Figure S20

Current smokers only.

Associations between high preference for a food preference item compared to low preference and all-cause mortality in current smokers only (n = 11,796). Only food preference items that were significant in the main analysis are included. Besides the Holm-adjusted p-value, the unadjusted p-value is included with its corresponding pointwise HR (95% CI). Models were adjusted for age, ethnic background, general health status, highest qualification, physical activity, and sex. Abbreviations: CI, Confidence interval; HR, Hazard ratio

Supplementary Tables

Table S1

All questionnaire items excluded from the analysis due to not being food preferences

Table S2

Food preference items included in analysis and number of participants included the respective models (n)

Table S3

Exact p-values of the main analysis and all sensitivity analyses

Table S4

Baseline characteristics after food preference group (high preference, 7-9))

Food preference groups were created according to the classification established by Concas and colleagues (3) who aggregated food items into preference groups based on similar liking ratings using cluster analysis and confirmed the resulting clusters through Cronbach's α reliability testing (3). The alcoholic beverages preference group consisted of preference for: bitter ale, lager, red wine, spirits, whisky, white wine. The cheese preference group consisted of preference for: blue cheese, hard cheese, soft cheese. The fish preference group consisted of preference for: baked/steamed fish, cod, fried battered fish, haddock, mackerel, prawns, salmon, sardines, shellfish, smoked fish, tinned tuna. The fruit preference group consisted of preference for: apples, bananas, cherries, dried fruit, fruit, grapefruit, kiwi fruit, lemons, melon, oranges, pears, plums, strawberries. The meat preference group consisted of preference for: bacon, barbequed/grilled meat, beef steak, bolognese sauce, burgers (meat), chicken, fried chicken, ham, lamb, pork chop, red meat, roast chicken, sausages (meat). The sweets preference group consisted of preference for: biscuits, cake, cake icing, cereal/granola bar, cheesecake, croissant, dark chocolate, honey, ice cream, jam, marzipan, milk chocolate, sweet foods and the vegetables preference group consisted of preference for: asparagus,

aubergine, avocados, beetroot, black olives, broad beans, broccoli, Brussel sprouts, cabbage, cauliflower, cucumber, fresh tomatoes, garlic, gherkins, green olives, horseradish/wasabi, lentils/beans, mushrooms, onions, potatoes, raw carrots, salad leaves, spinach, turnip (white), vegetables.

Table S5

Baseline characteristics after food preference group (low preference (1-3))

Food preference groups were created as described for table S4.

Table S6

Baseline Characteristics by FPQ Completion

This table depicts baseline characteristics for UK Biobank participants separated by FPQ completion.

References

1. Textor J. Drawing and Analyzing Causal DAGs with DAGitty. Nucleic Acids Res. 2015; (10):3220–7.

2. Textor J, van der Zander B, Gilthorpe MS, Liskiewicz M, Ellison GT. Robust causal inference using directed acyclic graphs: the R package 'dagitty'. Int J Epidemiol. 2016; 45(6):1887–94.

3. Concas MP, Catamo E, Biino G, Toniolo D, Gasparini P, Robino A. Factors associated with food liking and their relationship with metabolic traits in Italian cohorts. Food Qual Prefer 2019; 75:64–70.

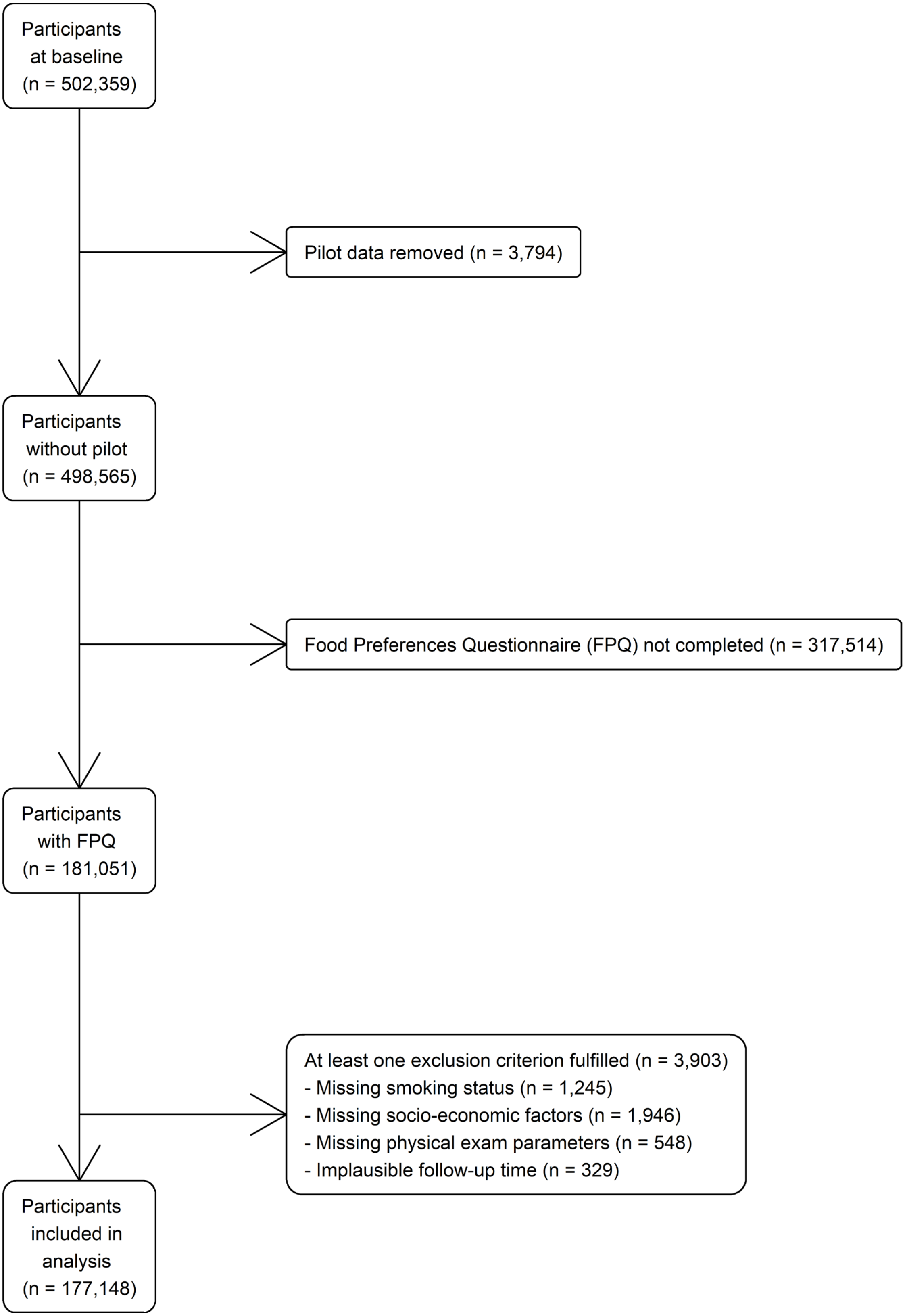

Figure S1. Flowchart of participant selection

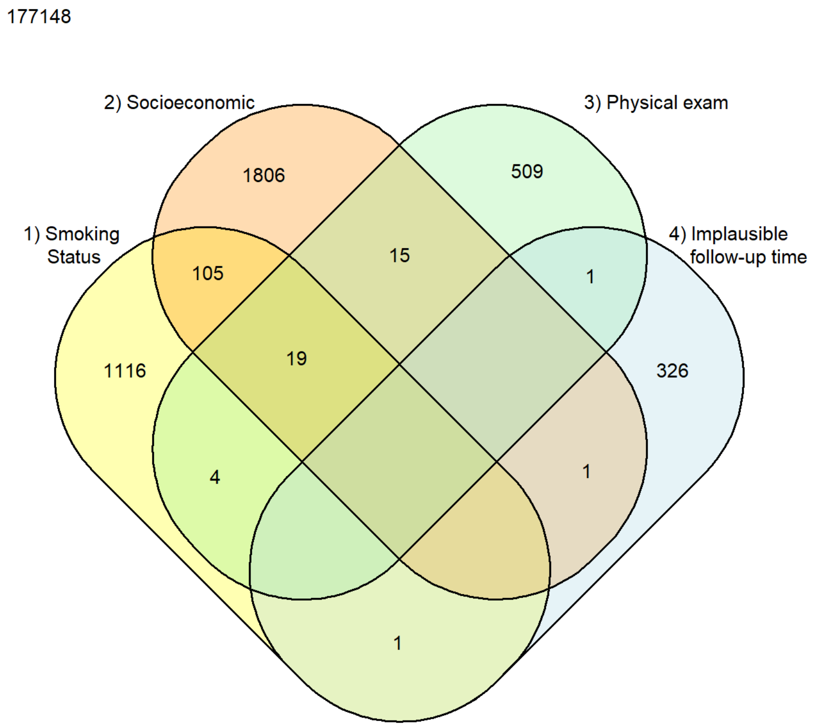

Figure S2. Venn Diagram of exclusion criteria

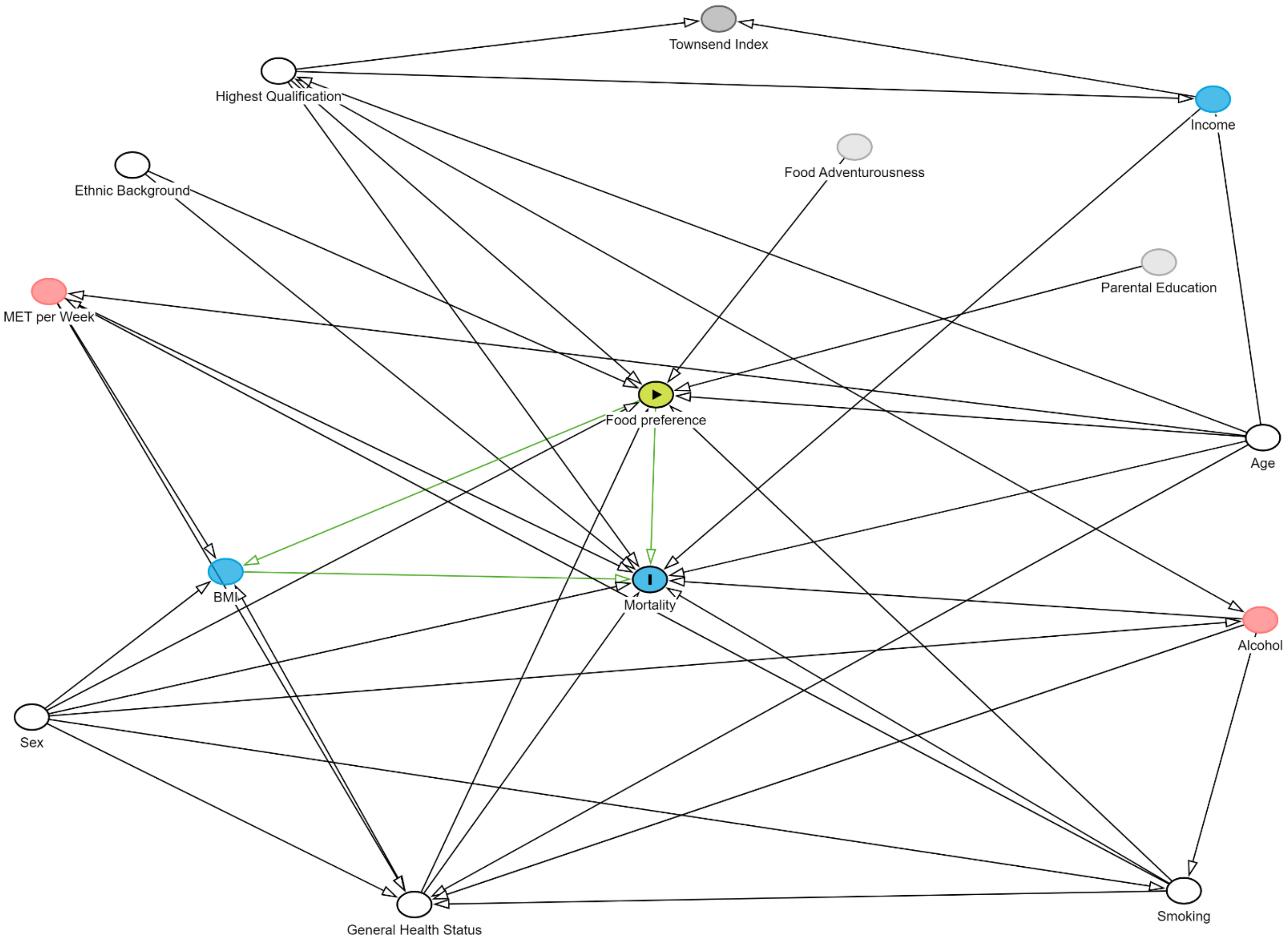

Figure S3. Diacyclic graph for covariate selection

Name	p-value (unadjusted)	p-value (Holm-adjusted)	HR (95% CI)
Adding salt to foods	0.1937	1.0000	1.06 (0.97 to 1.16)
Aniseed	0.2630	1.0000	0.95 (0.87 to 1.04)
Apple juice	0.0192	1.0000	1.13 (1.02 to 1.26)
Apples	0.0895	1.0000	0.85 (0.70 to 1.03)
Avocados	0.0025	0.2546	0.86 (0.78 to 0.95)
Bacon	0.1315	1.0000	1.10 (0.97 to 1.24)
Baked steamed fish	0.1505	1.0000	0.91 (0.81 to 1.03)
Bananas	0.0012	0.1215	0.79 (0.68 to 0.91)
Barbequed grilled meat	0.0199	1.0000	0.89 (0.80 to 0.98)
Beef steak	0.9234	1.0000	1.01 (0.90 to 1.12)
Beetroot	0.0011	0.1118	0.84 (0.75 to 0.93)
Biscuits	0.6772	1.0000	1.03 (0.90 to 1.17)
Bitter foods	0.0078	0.7266	0.87 (0.79 to 0.96)
Blue cheese	0.3406	1.0000	0.96 (0.88 to 1.04)
Bolognese sauce	0.1937	1.0000	0.92 (0.82 to 1.04)
Broad beans	0.0016	0.1682	0.85 (0.77 to 0.94)
Brown rice	< 0.0001	0.0726	0.81 (0.72 to 0.91)
Brussel sprouts	0.0358	1.0000	0.89 (0.80 to 0.99)
Burgers (meat)	0.3123	1.0000	1.05 (0.96 to 1.15)
Burn of spicy foods	0.0491	1.0000	0.92 (0.84 to 1.00)
Butter on bread	0.4407	1.0000	1.05 (0.93 to 1.19)
Cabbage	0.0885	1.0000	0.89 (0.77 to 1.02)
Cake	0.3248	1.0000	0.94 (0.83 to 1.06)
Cake icing	0.8242	1.0000	1.01 (0.93 to 1.10)
Capers	0.0072	0.6733	0.88 (0.80 to 0.96)
Cauliflower	0.4485	1.0000	0.94 (0.80 to 1.10)
Cereal granola bar	0.1525	1.0000	0.93 (0.84 to 1.03)
Cheesecake	0.3443	1.0000	0.95 (0.86 to 1.05)
Cherries	0.1587	1.0000	0.89 (0.75 to 1.05)
Chicken	0.8913	1.0000	1.01 (0.86 to 1.18)
Chilli pepper	0.0012	0.1251	0.87 (0.80 to 0.95)
Chips French fries	0.2003	1.0000	0.91 (0.79 to 1.05)
Cod	0.1831	1.0000	0.90 (0.77 to 1.05)
Coffee with sugar	0.0219	1.0000	1.10 (1.01 to 1.20)
Coffee without sugar	0.0055	0.5377	0.89 (0.82 to 0.97)
Cream	0.3604	1.0000	0.96 (0.87 to 1.05)
Croissant	0.0229	1.0000	0.89 (0.80 to 0.98)
Cucumber	0.5410	1.0000	0.97 (0.87 to 1.08)
Dairy products	0.4904	1.0000	0.94 (0.77 to 1.13)
Dark chocolate	0.2972	1.0000	0.94 (0.85 to 1.05)
Dried fruit	0.0189	1.0000	0.86 (0.75 to 0.97)
Eggs	0.0712	1.0000	0.84 (0.69 to 1.02)
Fatty foods	0.0245	1.0000	0.89 (0.81 to 0.99)
Fresh tomatoes	0.7655	1.0000	0.97 (0.81 to 1.17)
Fried chicken	0.6437	1.0000	1.02 (0.93 to 1.13)
Fried battered fish	0.2312	1.0000	1.07 (0.96 to 1.20)
Fruit	0.7420	1.0000	0.94 (0.64 to 1.38)
Gherkins	0.2319	1.0000	0.95 (0.87 to 1.03)
Globe artichoke	0.0988	1.0000	0.91 (0.82 to 1.02)
Haddock	0.5982	1.0000	1.04 (0.90 to 1.21)
Ham	0.0078	0.7266	1.19 (1.05 to 1.35)
Hard cheese	0.3546	1.0000	0.93 (0.79 to 1.09)
Herring	0.5469	1.0000	1.03 (0.94 to 1.12)
Honey	0.2330	1.0000	0.94 (0.85 to 1.04)

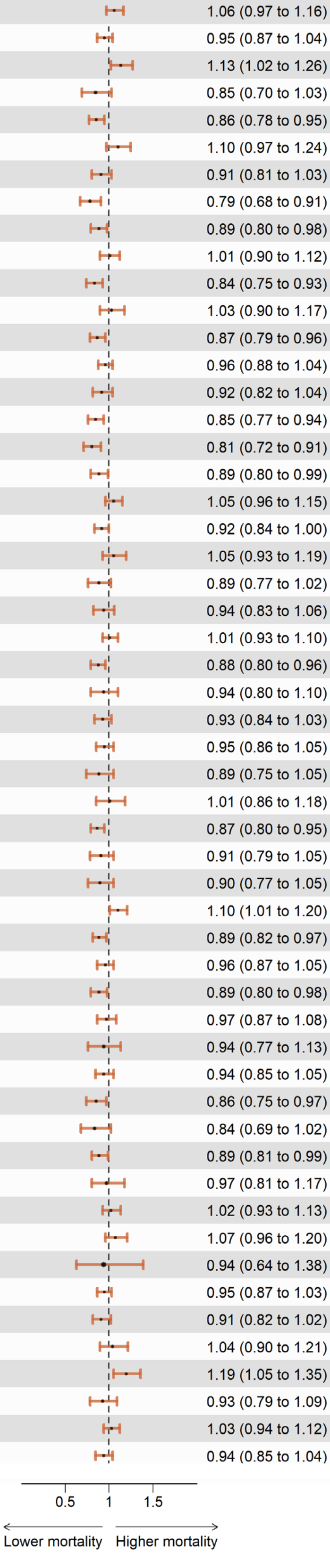

Figure S4. All food preference items with a Holm-adjusted p-value > 0.05

Name	p-value (unadjusted)	p-value (Holm-adjusted)	HR (95% CI)
Horseradish wasabi	0.2302	1.0000	0.95 (0.87 to 1.03)
Ice cream	0.3533	1.0000	1.07 (0.92 to 1.24)
Jam	0.9927	1.0000	1.00 (0.89 to 1.12)
Kiwi fruit	0.0171	1.0000	0.87 (0.77 to 0.97)
Lager	0.0098	0.8825	0.89 (0.81 to 0.97)
Lamb	0.3158	1.0000	1.06 (0.95 to 1.18)
Lemons	0.0139	1.0000	0.86 (0.77 to 0.97)
Liver	0.0932	1.0000	1.08 (0.99 to 1.17)
Mackerel	0.0120	1.0000	0.89 (0.81 to 0.97)
Marzipan	0.5405	1.0000	1.03 (0.95 to 1.11)
Mayonnaise	0.9559	1.0000	1.00 (0.91 to 1.10)
Melon	0.2994	1.0000	0.91 (0.77 to 1.08)
Milk chocolate	0.0193	1.0000	0.89 (0.81 to 0.98)
Orange juice	0.5227	1.0000	1.04 (0.92 to 1.17)
Oranges	0.1719	1.0000	0.89 (0.76 to 1.05)
Pasta	0.0149	1.0000	0.84 (0.73 to 0.97)
Pears	0.2726	1.0000	0.91 (0.77 to 1.08)
Pizza	0.0025	0.2505	0.85 (0.77 to 0.95)
Plums	0.1439	1.0000	0.88 (0.74 to 1.05)
Pollock	< 0.0001	0.0924	0.84 (0.75 to 0.93)
Pork chop	0.1571	1.0000	1.08 (0.97 to 1.19)
Porridge	0.1061	1.0000	0.91 (0.82 to 1.02)
Potato crisps	0.1175	1.0000	0.92 (0.82 to 1.02)
Potatoes	0.7488	1.0000	0.96 (0.75 to 1.23)
Prawns	0.4255	1.0000	0.96 (0.87 to 1.06)
Raw carrots	0.0039	0.3849	0.84 (0.74 to 0.94)
Red meat	0.2366	1.0000	1.07 (0.96 to 1.20)
Roast chicken	0.9979	1.0000	1.00 (0.85 to 1.17)
Salad dressing	0.1624	1.0000	0.93 (0.84 to 1.03)
Salami	0.7464	1.0000	1.01 (0.93 to 1.11)
Salmon	0.4363	1.0000	0.95 (0.84 to 1.08)
Salty foods	0.1720	1.0000	0.94 (0.86 to 1.03)
Salty pretzels	0.9047	1.0000	1.01 (0.91 to 1.12)
Sardines	0.4628	1.0000	1.04 (0.94 to 1.14)
Sausages (meat)	0.0163	1.0000	1.14 (1.02 to 1.26)
Savoury biscuits	0.4486	1.0000	1.06 (0.91 to 1.23)
Shellfish	0.2342	1.0000	0.95 (0.88 to 1.03)
Skimmed milk	0.0039	0.3849	0.88 (0.81 to 0.96)
Smoked fish	0.0828	1.0000	0.91 (0.82 to 1.01)
Soft cheese	0.9386	1.0000	1.00 (0.90 to 1.12)
Soy sauce	0.1972	1.0000	0.93 (0.84 to 1.04)
Soya milk	0.3394	1.0000	0.94 (0.83 to 1.07)
Strawberries	0.1621	1.0000	0.86 (0.70 to 1.06)
Sweet coffee house drinks	0.0065	0.6130	1.15 (1.04 to 1.28)
Sweet foods	0.4861	1.0000	0.97 (0.88 to 1.06)
Tea without sugar	0.0057	0.5460	0.88 (0.81 to 0.97)
Tomato ketchup	0.4251	1.0000	1.04 (0.95 to 1.13)
Turnip (white)	0.1080	1.0000	0.92 (0.84 to 1.02)
Vegetables	0.0083	0.7571	0.66 (0.48 to 0.90)
Vinegar	0.0952	1.0000	0.92 (0.83 to 1.01)
Whisky	0.0473	1.0000	0.92 (0.84 to 1.00)
White bread	0.0017	0.1777	1.15 (1.06 to 1.26)
White rice	0.8403	1.0000	0.99 (0.86 to 1.13)
Wholegrain breakfast cereal	0.0212	1.0000	0.86 (0.76 to 0.98)

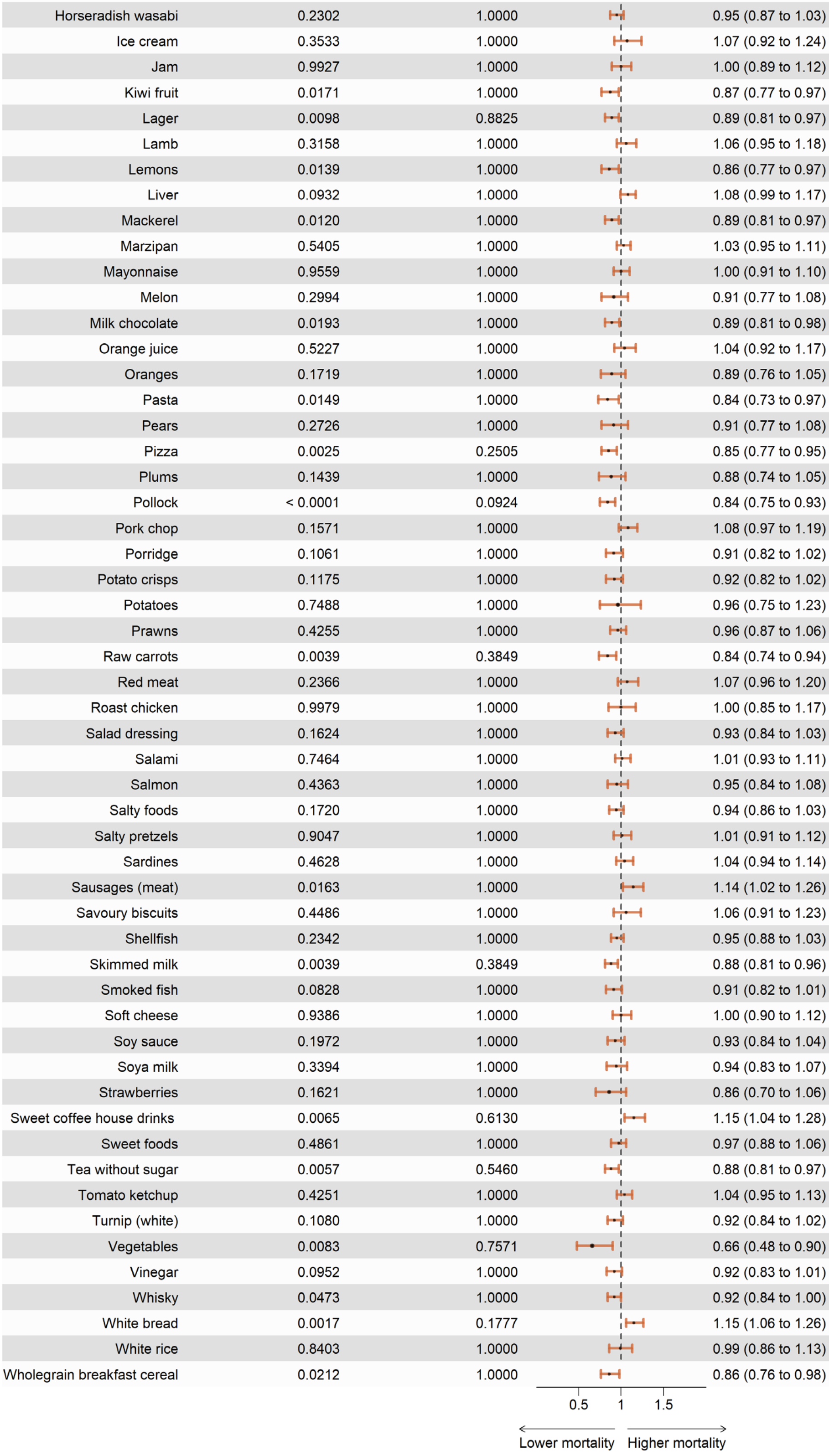

Figure S4. All food preference items with a Holm-adjusted p-value > 0.05 (continued)

Name	p-value (unadjusted)	p-value (Holm-adjusted)	HR (95% CI)
Asparagus	< 0.0001	< 0.0001	0.69 (0.61 to 0.78)
Aubergine	< 0.0001	0.0222	0.82 (0.74 to 0.91)
Bell pepper	< 0.0001	0.0032	0.77 (0.69 to 0.87)
Bitter ale	< 0.0001	0.0326	0.83 (0.75 to 0.92)
Black olives	0.0046	0.4772	0.88 (0.80 to 0.96)
Black pepper	< 0.0001	< 0.0001	0.73 (0.64 to 0.83)
Broccoli	< 0.0001	0.0193	0.75 (0.64 to 0.87)
Butternut squash	< 0.0001	< 0.0001	0.76 (0.68 to 0.85)
Coriander	< 0.0001	0.0538	0.81 (0.72 to 0.91)
Corn flakes	< 0.0001	0.0476	1.22 (1.09 to 1.36)
Curry	< 0.0001	0.0575	0.82 (0.74 to 0.92)
Diet fizzy drinks	0.0012	0.1309	1.20 (1.07 to 1.33)
Extra virgin olive oil	< 0.0001	< 0.0001	0.69 (0.58 to 0.81)
Garlic	0.0169	1.0000	0.87 (0.77 to 0.97)
Goats cheese	< 0.0001	0.0757	0.84 (0.76 to 0.93)
Grapefruit	< 0.0001	0.0948	0.85 (0.77 to 0.93)
Green olives	0.0039	0.4139	0.87 (0.80 to 0.96)
Lentils and beans	< 0.0001	0.0014	0.73 (0.64 to 0.84)
Mushrooms	< 0.0001	0.0603	0.75 (0.64 to 0.88)
Onions	0.0010	0.1141	0.76 (0.65 to 0.90)
Plain yogurt	0.0011	0.1202	0.84 (0.75 to 0.93)
Red wine	< 0.0001	0.0052	0.81 (0.74 to 0.90)
Regular fizzy drinks	< 0.0001	< 0.0001	1.40 (1.24 to 1.58)
Salad leaves	< 0.0001	0.0582	0.74 (0.63 to 0.88)
Spicy foods	< 0.0001	0.0463	0.84 (0.76 to 0.92)
Spinach	< 0.0001	0.0727	0.81 (0.72 to 0.91)
Spirits	< 0.0001	0.0216	0.84 (0.77 to 0.92)
Tea with sugar	< 0.0001	0.0463	1.21 (1.09 to 1.34)
Tinned tuna	< 0.0001	0.0024	0.79 (0.71 to 0.88)
White wine	< 0.0001	0.0030	0.80 (0.73 to 0.89)
Whole milk	< 0.0001	0.0042	1.22 (1.11 to 1.34)
Wholemeal bread	< 0.0001	0.0380	0.70 (0.58 to 0.85)

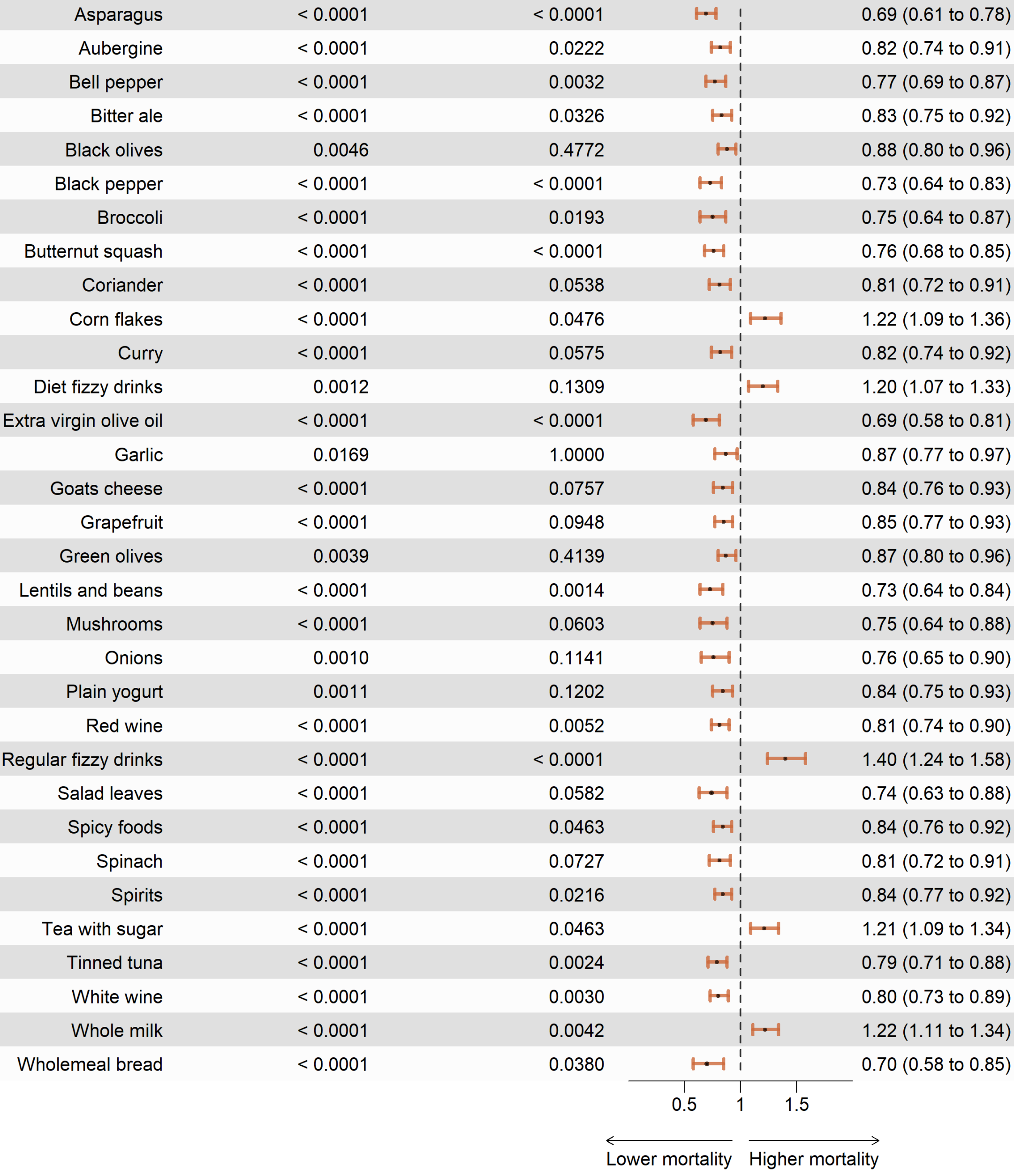

Figure S5. Participants with unintentional weight loss removed

Name	p-value (unadjusted)	p-value (Holm-adjusted)	HR (95% CI)
Asparagus	< 0.0001	< 0.0001	0.68 (0.60 to 0.77)
Aubergine	< 0.0001	< 0.0001	0.77 (0.69 to 0.85)
Bell pepper	0.0028	0.3009	0.83 (0.73 to 0.94)
Bitter ale	< 0.0001	0.0400	0.83 (0.75 to 0.92)
Black olives	0.0037	0.3867	0.87 (0.79 to 0.96)
Black pepper	< 0.0001	< 0.0001	0.72 (0.63 to 0.82)
Broccoli	< 0.0001	0.0010	0.71 (0.62 to 0.83)
Butternut squash	< 0.0001	< 0.0001	0.76 (0.68 to 0.85)
Coriander	0.0018	0.2033	0.83 (0.73 to 0.93)
Corn flakes	0.0136	1.0000	1.15 (1.03 to 1.28)
Curry	< 0.0001	0.0261	0.81 (0.73 to 0.91)
Diet fizzy drinks	0.0017	0.1948	1.19 (1.07 to 1.33)
Extra virgin olive oil	< 0.0001	< 0.0001	0.69 (0.58 to 0.81)
Garlic	0.0020	0.2240	0.83 (0.74 to 0.93)
Goats cheese	< 0.0001	0.0261	0.83 (0.75 to 0.91)
Grapefruit	0.0022	0.2451	0.86 (0.78 to 0.95)
Green olives	0.0017	0.1978	0.86 (0.79 to 0.95)
Lentils and beans	< 0.0001	0.0031	0.74 (0.64 to 0.85)
Mushrooms	0.0017	0.1948	0.77 (0.65 to 0.91)
Onions	0.0015	0.1840	0.76 (0.65 to 0.90)
Plain yogurt	0.0063	0.6218	0.86 (0.77 to 0.96)
Red wine	< 0.0001	0.0391	0.83 (0.75 to 0.92)
Regular fizzy drinks	< 0.0001	< 0.0001	1.35 (1.19 to 1.52)
Salad leaves	< 0.0001	0.0130	0.72 (0.60 to 0.85)
Spicy foods	< 0.0001	0.0252	0.83 (0.75 to 0.91)
Spinach	< 0.0001	0.0067	0.78 (0.69 to 0.88)
Spirits	< 0.0001	0.0031	0.82 (0.75 to 0.90)
Tea with sugar	< 0.0001	< 0.0001	1.29 (1.16 to 1.43)
Tinned tuna	< 0.0001	0.0423	0.82 (0.74 to 0.91)
White wine	< 0.0001	0.0042	0.81 (0.73 to 0.89)
Whole milk	0.0018	0.2033	1.16 (1.06 to 1.28)
Wholemeal bread	< 0.0001	0.0416	0.70 (0.58 to 0.85)

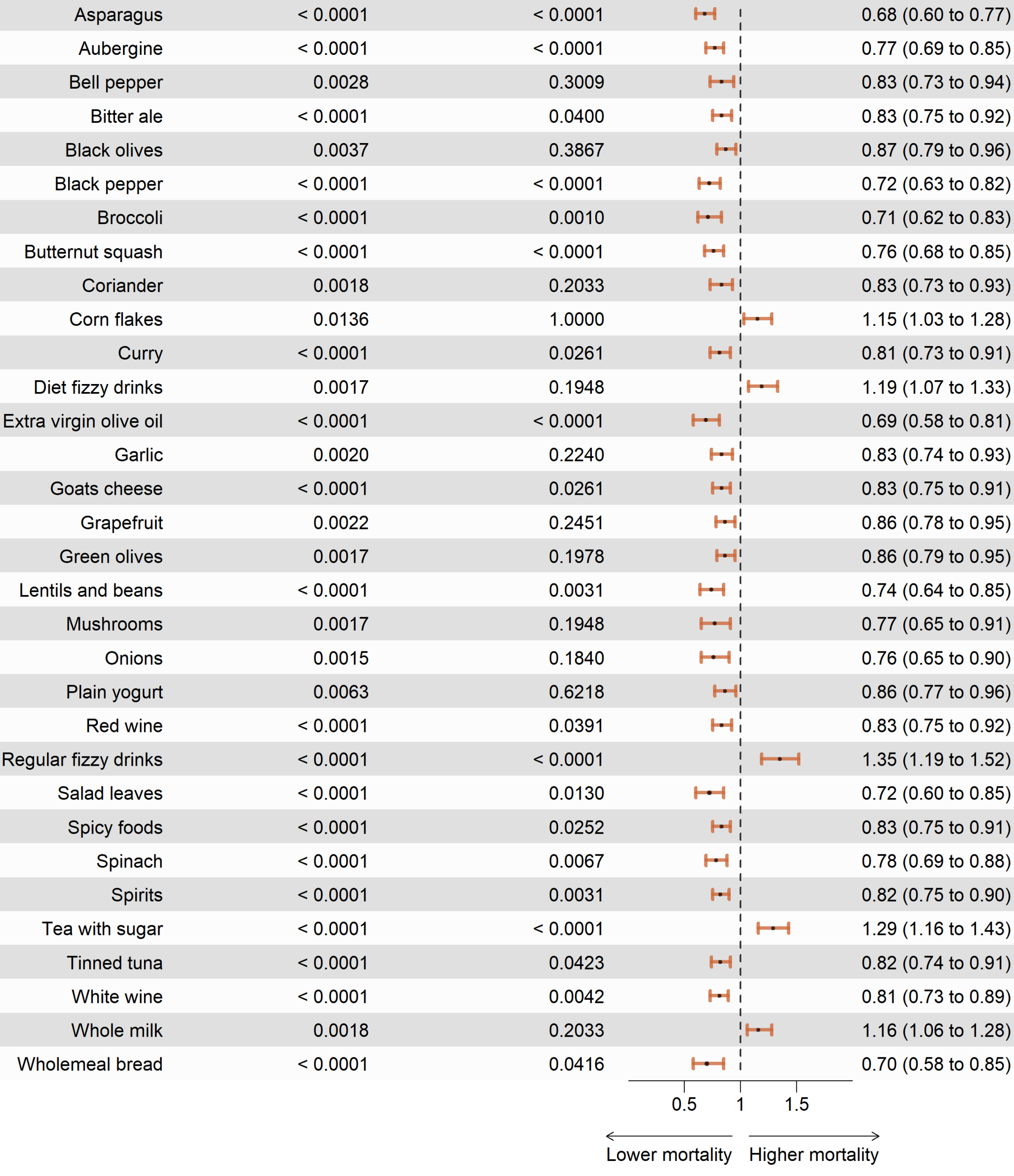

Figure S6. Landmark analysis (up to 1 year follow-up excluded)

Name	p-value (unadjusted)	p-value (Holm-adjusted)	HR (95% CI)
Asparagus	< 0.0001	< 0.0001	0.71 (0.64 to 0.80)
Aubergine	< 0.0001	0.0177	0.82 (0.75 to 0.91)
Bell pepper	< 0.0001	0.0501	0.81 (0.73 to 0.91)
Bitter ale	< 0.0001	0.0138	0.83 (0.75 to 0.91)
Black olives	0.0030	0.3209	0.88 (0.80 to 0.96)
Black pepper	< 0.0001	< 0.0001	0.72 (0.64 to 0.81)
Broccoli	< 0.0001	< 0.0001	0.71 (0.62 to 0.82)
Butternut squash	< 0.0001	< 0.0001	0.77 (0.69 to 0.85)
Coriander	0.0024	0.2624	0.84 (0.75 to 0.94)
Corn flakes	0.0011	0.1261	1.19 (1.07 to 1.32)
Curry	< 0.0001	0.0029	0.80 (0.72 to 0.89)
Diet fizzy drinks	0.1402	1.0000	1.08 (0.97 to 1.20)
Extra virgin olive oil	< 0.0001	0.0020	0.71 (0.61 to 0.83)
Garlic	< 0.0001	0.0488	0.82 (0.73 to 0.91)
Goats cheese	< 0.0001	0.0468	0.84 (0.77 to 0.93)
Grapefruit	0.0048	0.4973	0.88 (0.80 to 0.96)
Green olives	< 0.0001	0.1066	0.86 (0.79 to 0.94)
Lentils and beans	< 0.0001	0.0408	0.78 (0.68 to 0.89)
Mushrooms	< 0.0001	0.0177	0.75 (0.64 to 0.87)
Onions	< 0.0001	0.0028	0.72 (0.62 to 0.84)
Plain yogurt	0.0026	0.2819	0.85 (0.77 to 0.95)
Red wine	0.0011	0.1293	0.86 (0.78 to 0.94)
Regular fizzy drinks	< 0.0001	< 0.0001	1.40 (1.24 to 1.57)
Salad leaves	< 0.0001	0.0314	0.74 (0.63 to 0.87)
Spicy foods	< 0.0001	0.0023	0.82 (0.74 to 0.89)
Spinach	< 0.0001	0.0076	0.79 (0.71 to 0.89)
Spirits	< 0.0001	0.0014	0.82 (0.75 to 0.90)
Tea with sugar	< 0.0001	< 0.0001	1.27 (1.15 to 1.41)
Tinned tuna	< 0.0001	0.0502	0.83 (0.75 to 0.92)
White wine	< 0.0001	0.0991	0.85 (0.77 to 0.93)
Whole milk	< 0.0001	0.0231	1.18 (1.08 to 1.29)
Wholemeal bread	< 0.0001	0.0044	0.68 (0.57 to 0.82)

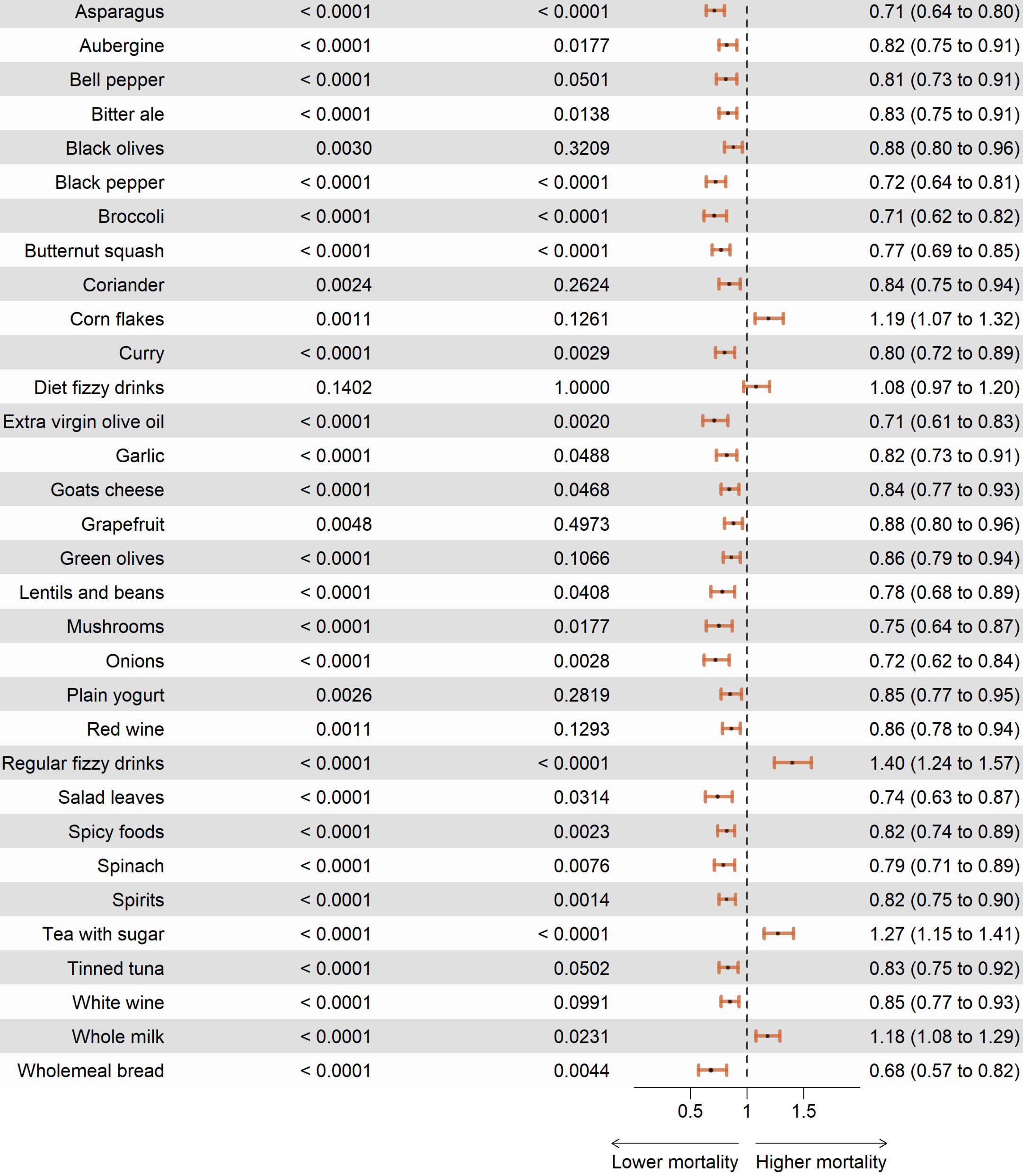

Figure S7. Participants with diabetes mellitus excluded

Name	p-value (unadjusted)	p-value (Holm-adjusted)	HR (95% CI)
Asparagus	< 0.0001	< 0.0001	0.73 (0.65 to 0.82)
Aubergine	< 0.0001	0.0104	0.82 (0.74 to 0.90)
Bell pepper	< 0.0001	0.0068	0.79 (0.71 to 0.89)
Bitter ale	< 0.0001	0.0179	0.83 (0.75 to 0.91)
Black olives	0.0012	0.1352	0.87 (0.80 to 0.95)
Black pepper	< 0.0001	< 0.0001	0.72 (0.64 to 0.81)
Broccoli	< 0.0001	< 0.0001	0.70 (0.61 to 0.81)
Butternut squash	< 0.0001	< 0.0001	0.75 (0.68 to 0.84)
Coriander	0.0027	0.2829	0.84 (0.75 to 0.94)
Corn flakes	< 0.0001	0.0963	1.19 (1.07 to 1.32)
Curry	< 0.0001	0.0025	0.80 (0.72 to 0.89)
Diet fizzy drinks	0.0182	1.0000	1.13 (1.02 to 1.25)
Extra virgin olive oil	< 0.0001	< 0.0001	0.69 (0.59 to 0.80)
Garlic	< 0.0001	0.0068	0.80 (0.71 to 0.89)
Goats cheese	< 0.0001	0.0355	0.84 (0.77 to 0.92)
Grapefruit	< 0.0001	0.0513	0.85 (0.78 to 0.93)
Green olives	< 0.0001	0.0666	0.86 (0.79 to 0.94)
Lentils and beans	< 0.0001	0.0524	0.79 (0.69 to 0.90)
Mushrooms	< 0.0001	0.0048	0.73 (0.63 to 0.85)
Onions	< 0.0001	0.0062	0.73 (0.62 to 0.85)
Plain yogurt	0.0015	0.1664	0.85 (0.77 to 0.94)
Red wine	< 0.0001	0.0637	0.85 (0.77 to 0.93)
Regular fizzy drinks	< 0.0001	< 0.0001	1.31 (1.17 to 1.47)
Salad leaves	< 0.0001	0.0036	0.71 (0.61 to 0.84)
Spicy foods	< 0.0001	0.0027	0.82 (0.74 to 0.90)
Spinach	< 0.0001	< 0.0001	0.76 (0.68 to 0.85)
Spirits	< 0.0001	< 0.0001	0.81 (0.75 to 0.89)
Tea with sugar	< 0.0001	0.0024	1.24 (1.13 to 1.38)
Tinned tuna	< 0.0001	0.0177	0.82 (0.74 to 0.91)
White wine	0.0010	0.1140	0.85 (0.77 to 0.94)
Whole milk	< 0.0001	0.0281	1.18 (1.08 to 1.29)
Wholemeal bread	< 0.0001	0.0018	0.67 (0.56 to 0.81)

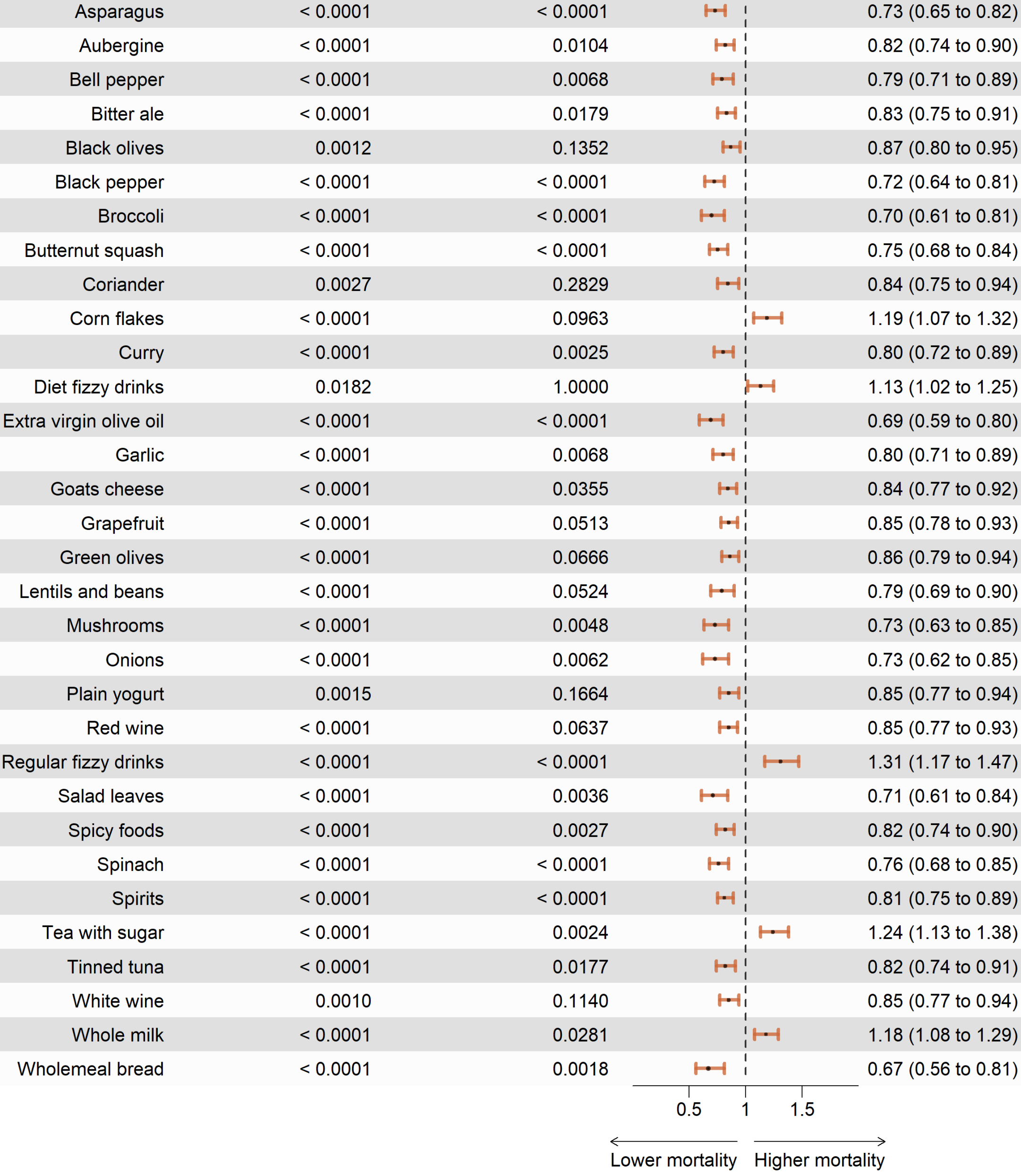

Figure S8. Participants with GFR ≥ 60 at baseline assessment

Name	p-value (unadjusted)	p-value (Holm-adjusted)	HR (95% CI)
Asparagus	< 0.0001	< 0.0001	0.72 (0.64 to 0.81)
Aubergine	< 0.0001	0.0063	0.82 (0.74 to 0.90)
Bell pepper	< 0.0001	0.0089	0.80 (0.71 to 0.89)
Bitter ale	< 0.0001	0.0022	0.81 (0.74 to 0.89)
Black olives	0.0016	0.1739	0.87 (0.80 to 0.95)
Black pepper	< 0.0001	< 0.0001	0.72 (0.64 to 0.81)
Broccoli	< 0.0001	< 0.0001	0.72 (0.63 to 0.82)
Butternut squash	< 0.0001	< 0.0001	0.77 (0.69 to 0.85)
Coriander	0.0026	0.2602	0.84 (0.75 to 0.94)
Corn flakes	< 0.0001	0.0180	1.22 (1.10 to 1.35)
Curry	< 0.0001	0.0078	0.81 (0.73 to 0.90)
Diet fizzy drinks	0.0012	0.1334	1.18 (1.07 to 1.31)
Extra virgin olive oil	< 0.0001	< 0.0001	0.67 (0.58 to 0.77)
Garlic	< 0.0001	0.0044	0.80 (0.71 to 0.89)
Goats cheese	< 0.0001	0.0261	0.84 (0.77 to 0.92)
Grapefruit	0.0022	0.2272	0.87 (0.79 to 0.95)
Green olives	0.0017	0.1813	0.87 (0.80 to 0.95)
Lentils and beans	< 0.0001	0.0098	0.77 (0.67 to 0.88)
Mushrooms	< 0.0001	0.0176	0.75 (0.65 to 0.87)
Onions	< 0.0001	0.0138	0.74 (0.63 to 0.86)
Plain yogurt	0.0014	0.1580	0.85 (0.77 to 0.94)
Red wine	< 0.0001	0.0529	0.85 (0.77 to 0.93)
Regular fizzy drinks	< 0.0001	< 0.0001	1.39 (1.24 to 1.56)
Salad leaves	< 0.0001	0.0288	0.74 (0.64 to 0.87)
Spicy foods	< 0.0001	0.0096	0.83 (0.76 to 0.91)
Spinach	< 0.0001	0.0058	0.79 (0.71 to 0.89)
Spirits	< 0.0001	0.0010	0.82 (0.75 to 0.90)
Tea with sugar	< 0.0001	0.0028	1.24 (1.12 to 1.37)
Tinned tuna	< 0.0001	< 0.0001	0.78 (0.71 to 0.86)
White wine	< 0.0001	0.0261	0.84 (0.76 to 0.92)
Whole milk	< 0.0001	0.0261	1.18 (1.08 to 1.29)
Wholemeal bread	< 0.0001	0.0036	0.69 (0.58 to 0.82)

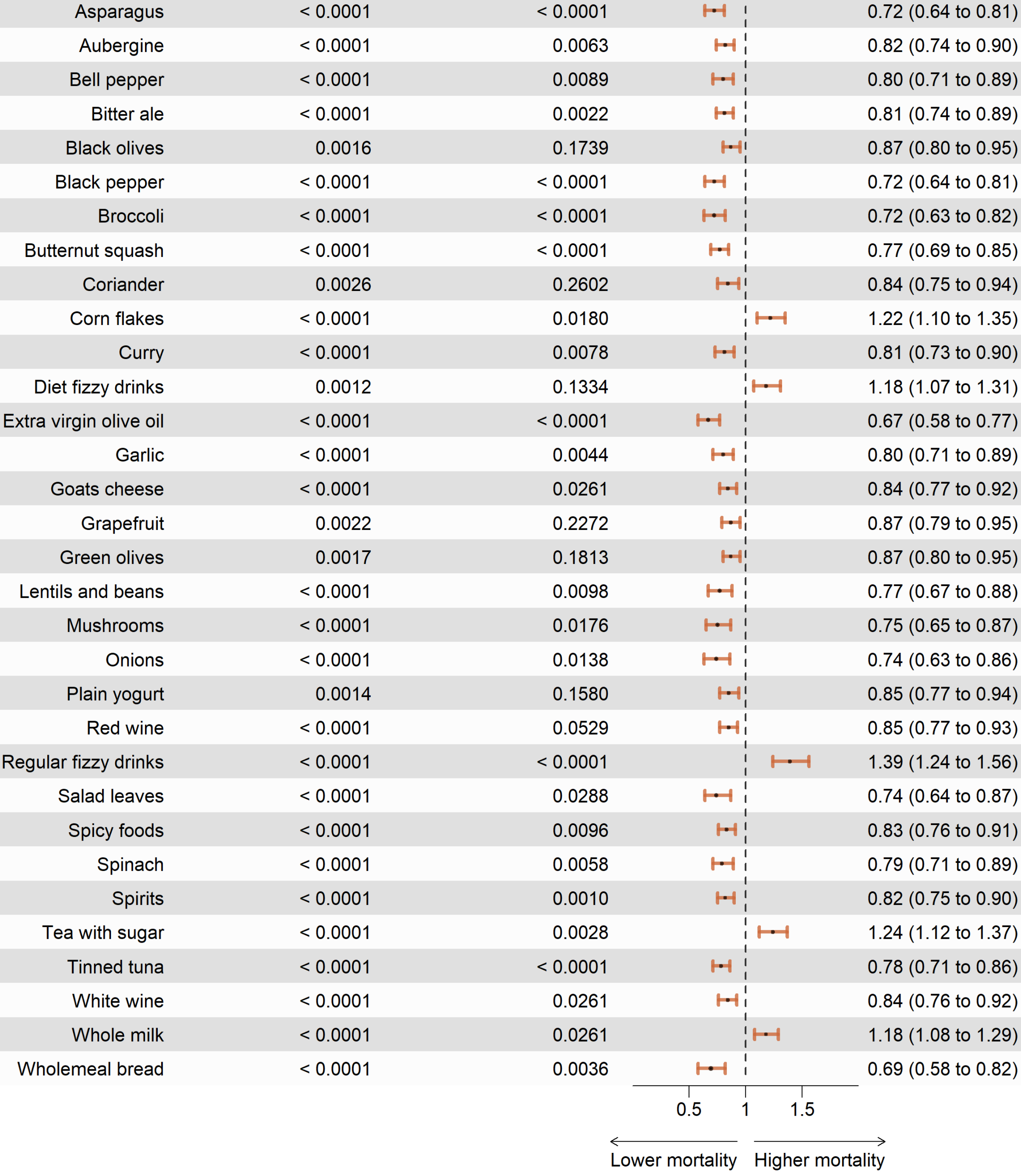

Figure S9. Participants without history of any psychiatric disease

Name	p-value (unadjusted)	p-value (Holm-adjusted)	HR (95% CI)
Asparagus	< 0.0001	< 0.0001	0.75 (0.66 to 0.84)
Aubergine	< 0.0001	0.0073	0.81 (0.73 to 0.90)
Bell pepper	0.0011	0.1212	0.82 (0.73 to 0.92)
Bitter ale	< 0.0001	0.0289	0.83 (0.75 to 0.92)
Black olives	0.0016	0.1753	0.87 (0.79 to 0.95)
Black pepper	< 0.0001	0.0018	0.76 (0.67 to 0.86)
Broccoli	< 0.0001	< 0.0001	0.71 (0.62 to 0.82)
Butternut squash	< 0.0001	< 0.0001	0.77 (0.69 to 0.85)
Coriander	0.0060	0.6265	0.85 (0.76 to 0.95)
Corn flakes	< 0.0001	0.0642	1.21 (1.09 to 1.34)
Curry	< 0.0001	0.0137	0.81 (0.73 to 0.90)
Diet fizzy drinks	0.0030	0.3320	1.17 (1.05 to 1.30)
Extra virgin olive oil	< 0.0001	0.0061	0.72 (0.62 to 0.84)
Garlic	< 0.0001	0.0943	0.82 (0.73 to 0.92)
Goats cheese	< 0.0001	0.0776	0.85 (0.77 to 0.93)
Grapefruit	< 0.0001	0.1080	0.85 (0.78 to 0.94)
Green olives	0.0048	0.5109	0.88 (0.81 to 0.96)
Lentils and beans	< 0.0001	0.0244	0.77 (0.67 to 0.88)
Mushrooms	< 0.0001	0.0776	0.76 (0.65 to 0.89)
Onions	< 0.0001	0.0037	0.72 (0.61 to 0.84)
Plain yogurt	< 0.0001	0.0782	0.83 (0.75 to 0.93)
Red wine	< 0.0001	0.1014	0.85 (0.77 to 0.94)
Regular fizzy drinks	< 0.0001	< 0.0001	1.39 (1.24 to 1.56)
Salad leaves	< 0.0001	0.1014	0.76 (0.64 to 0.89)
Spicy foods	< 0.0001	0.0423	0.84 (0.76 to 0.92)
Spinach	< 0.0001	0.0058	0.79 (0.70 to 0.88)
Spirits	< 0.0001	0.0018	0.82 (0.75 to 0.90)
Tea with sugar	< 0.0001	0.0114	1.23 (1.11 to 1.36)
Tinned tuna	< 0.0001	0.0013	0.79 (0.72 to 0.88)
White wine	< 0.0001	0.0372	0.84 (0.76 to 0.92)
Whole milk	< 0.0001	0.0895	1.17 (1.07 to 1.28)
Wholemeal bread	< 0.0001	0.0215	0.70 (0.58 to 0.84)

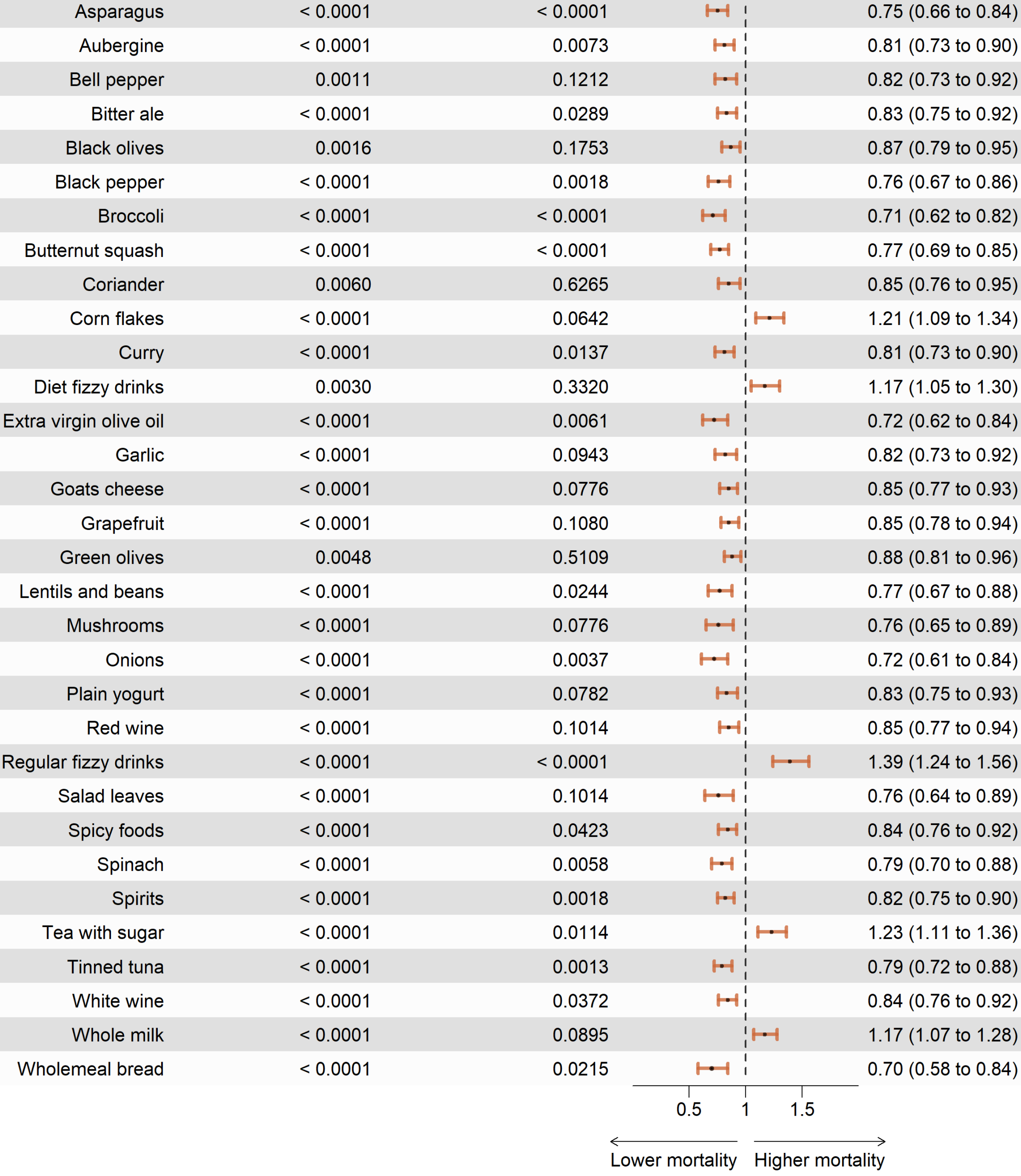

Figure S10. Participants with history of cancer excluded

Name	p-value (unadjusted)	p-value (Holm-adjusted)	HR (95% CI)
Asparagus	< 0.0001	< 0.0001	0.71 (0.63 to 0.80)
Aubergine	< 0.0001	0.0111	0.82 (0.74 to 0.90)
Bell pepper	< 0.0001	0.0241	0.80 (0.72 to 0.90)
Bitter ale	< 0.0001	0.0325	0.83 (0.76 to 0.92)
Black olives	0.0012	0.1384	0.87 (0.79 to 0.95)
Black pepper	< 0.0001	< 0.0001	0.71 (0.63 to 0.80)
Broccoli	< 0.0001	0.0040	0.74 (0.64 to 0.85)
Butternut squash	< 0.0001	< 0.0001	0.76 (0.68 to 0.85)
Coriander	0.0028	0.2971	0.84 (0.75 to 0.94)
Corn flakes	< 0.0001	0.0389	1.21 (1.09 to 1.34)
Curry	< 0.0001	0.0345	0.82 (0.74 to 0.92)
Diet fizzy drinks	0.0059	0.6051	1.16 (1.04 to 1.28)
Extra virgin olive oil	< 0.0001	< 0.0001	0.70 (0.60 to 0.81)
Garlic	< 0.0001	0.0072	0.80 (0.71 to 0.89)
Goats cheese	< 0.0001	0.0514	0.84 (0.77 to 0.93)
Grapefruit	0.0022	0.2468	0.87 (0.79 to 0.95)
Green olives	< 0.0001	0.0725	0.86 (0.79 to 0.94)
Lentils and beans	0.0012	0.1358	0.80 (0.70 to 0.91)
Mushrooms	< 0.0001	0.0240	0.75 (0.64 to 0.87)
Onions	< 0.0001	0.0049	0.72 (0.62 to 0.84)
Plain yogurt	< 0.0001	0.0570	0.83 (0.75 to 0.92)
Red wine	0.0020	0.2260	0.86 (0.79 to 0.95)
Regular fizzy drinks	< 0.0001	< 0.0001	1.38 (1.23 to 1.56)
Salad leaves	0.0012	0.1404	0.76 (0.64 to 0.90)
Spicy foods	< 0.0001	0.0058	0.82 (0.75 to 0.90)
Spinach	< 0.0001	< 0.0001	0.76 (0.68 to 0.85)
Spirits	< 0.0001	< 0.0001	0.81 (0.74 to 0.88)
Tea with sugar	< 0.0001	0.0241	1.21 (1.10 to 1.34)
Tinned tuna	0.0013	0.1432	0.84 (0.76 to 0.94)
White wine	< 0.0001	0.1031	0.85 (0.77 to 0.93)
Whole milk	< 0.0001	0.0023	1.22 (1.11 to 1.33)
Wholemeal bread	< 0.0001	0.0061	0.69 (0.57 to 0.82)

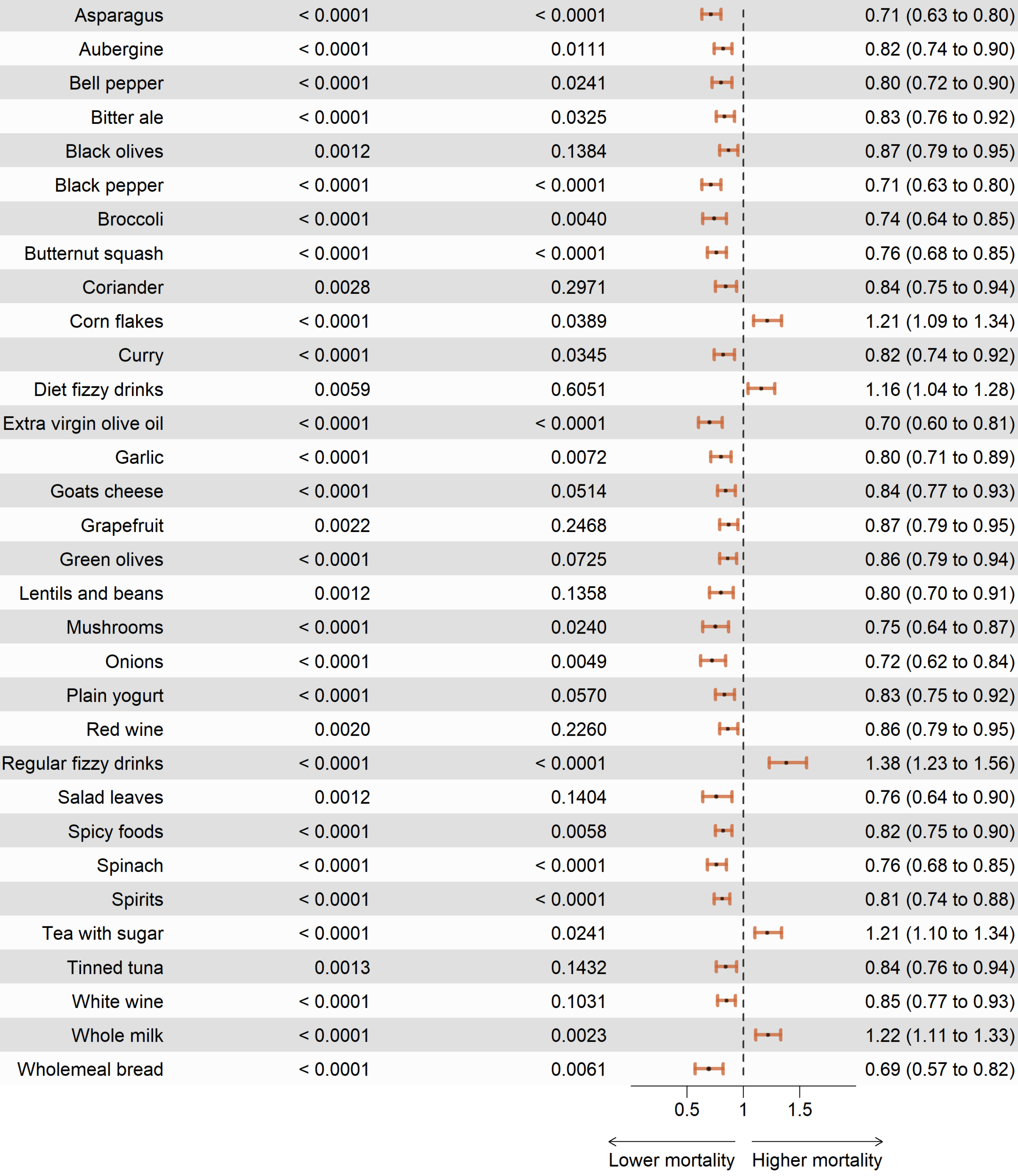

Figure S11. Participants with history of cardiovascular disease excluded

Name	p-value (unadjusted)	p-value (Holm-adjusted)	HR (95% CI)
Asparagus	< 0.0001	< 0.0001	0.69 (0.61 to 0.79)
Aubergine	< 0.0001	0.0209	0.80 (0.71 to 0.90)
Bell pepper	0.0061	0.6237	0.83 (0.72 to 0.95)
Bitter ale	0.0046	0.4990	0.85 (0.76 to 0.95)
Black olives	< 0.0001	< 0.0001	0.80 (0.72 to 0.88)
Black pepper	< 0.0001	< 0.0001	0.70 (0.60 to 0.80)
Broccoli	< 0.0001	0.0107	0.73 (0.62 to 0.85)
Butternut squash	< 0.0001	< 0.0001	0.73 (0.65 to 0.83)
Coriander	0.0011	0.1336	0.81 (0.71 to 0.92)
Corn flakes	0.0045	0.4852	1.19 (1.06 to 1.35)
Curry	0.0052	0.5578	0.84 (0.74 to 0.95)
Diet fizzy drinks	0.0219	1.0000	1.15 (1.02 to 1.29)
Extra virgin olive oil	< 0.0001	< 0.0001	0.65 (0.55 to 0.77)
Garlic	0.0018	0.2073	0.81 (0.71 to 0.93)
Goats cheese	0.0044	0.4800	0.85 (0.76 to 0.95)
Grapefruit	0.0040	0.4429	0.86 (0.77 to 0.95)
Green olives	< 0.0001	0.0028	0.81 (0.73 to 0.89)
Lentils and beans	< 0.0001	0.0095	0.74 (0.63 to 0.86)
Mushrooms	< 0.0001	0.0243	0.72 (0.61 to 0.86)
Onions	0.0011	0.1322	0.74 (0.61 to 0.88)
Plain yogurt	< 0.0001	0.0158	0.80 (0.71 to 0.89)
Red wine	0.0124	1.0000	0.87 (0.78 to 0.97)
Regular fizzy drinks	< 0.0001	< 0.0001	1.37 (1.20 to 1.56)
Salad leaves	< 0.0001	0.0634	0.72 (0.60 to 0.87)
Spicy foods	< 0.0001	0.0503	0.82 (0.74 to 0.92)
Spinach	< 0.0001	0.0297	0.78 (0.69 to 0.89)
Spirits	< 0.0001	0.0660	0.84 (0.76 to 0.93)
Tea with sugar	0.0019	0.2249	1.20 (1.07 to 1.35)
Tinned tuna	< 0.0001	< 0.0001	0.76 (0.68 to 0.86)
White wine	0.0088	0.8482	0.86 (0.77 to 0.96)
Whole milk	0.0053	0.5578	1.16 (1.04 to 1.28)
Wholemeal bread	0.0036	0.4094	0.73 (0.59 to 0.90)

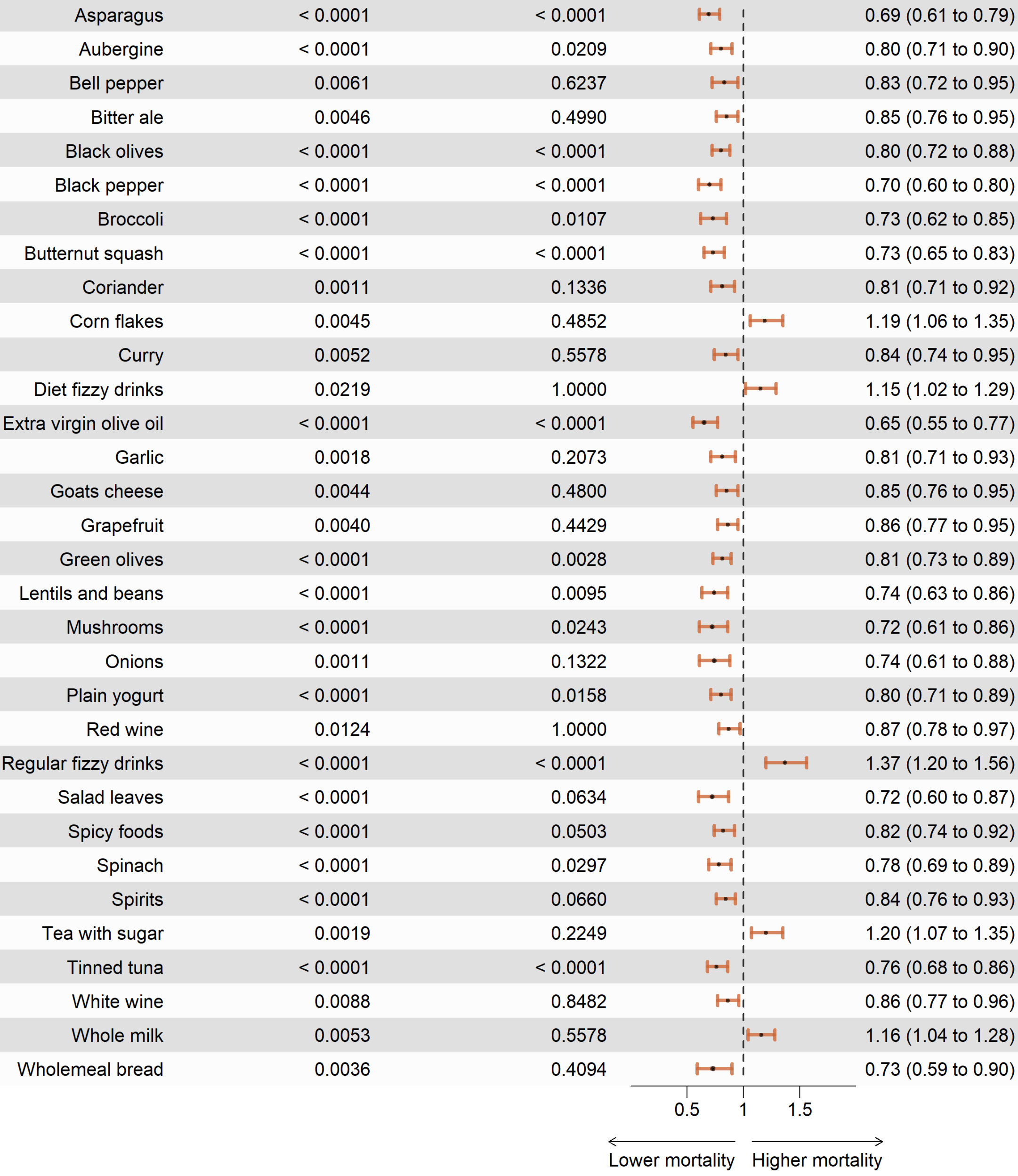

Figure S12. Participants not taking any vitamin or mineral supplements

Name	p-value (unadjusted)	p-value (Holm-adjusted)	HR (95% CI)
Asparagus	< 0.0001	< 0.0001	0.71 (0.63 to 0.79)
Aubergine	< 0.0001	0.0022	0.81 (0.74 to 0.89)
Bell pepper	< 0.0001	0.0060	0.80 (0.72 to 0.89)
Bitter ale	< 0.0001	0.0021	0.82 (0.75 to 0.90)
Black olives	< 0.0001	0.0544	0.86 (0.80 to 0.94)
Black pepper	< 0.0001	< 0.0001	0.72 (0.64 to 0.80)
Broccoli	< 0.0001	< 0.0001	0.70 (0.62 to 0.80)
Butternut squash	< 0.0001	< 0.0001	0.76 (0.69 to 0.84)
Coriander	< 0.0001	0.0743	0.83 (0.75 to 0.92)
Corn flakes	< 0.0001	0.0743	1.19 (1.08 to 1.31)
Curry	< 0.0001	0.0032	0.81 (0.74 to 0.89)
Diet fizzy drinks	0.0032	0.3150	1.16 (1.05 to 1.28)
Extra virgin olive oil	< 0.0001	< 0.0001	0.68 (0.59 to 0.79)
Garlic	< 0.0001	0.0020	0.79 (0.72 to 0.88)
Goats cheese	< 0.0001	0.0175	0.84 (0.77 to 0.92)
Grapefruit	0.0014	0.1452	0.87 (0.80 to 0.95)
Green olives	< 0.0001	0.0481	0.86 (0.79 to 0.94)
Lentils and beans	< 0.0001	0.0037	0.76 (0.67 to 0.87)
Mushrooms	< 0.0001	0.0150	0.76 (0.65 to 0.87)
Onions	< 0.0001	0.0019	0.72 (0.63 to 0.84)
Plain yogurt	< 0.0001	0.0472	0.84 (0.76 to 0.93)
Red wine	< 0.0001	0.0257	0.85 (0.77 to 0.92)
Regular fizzy drinks	< 0.0001	< 0.0001	1.36 (1.22 to 1.52)
Salad leaves	< 0.0001	0.0045	0.73 (0.63 to 0.85)
Spicy foods	< 0.0001	0.0020	0.82 (0.75 to 0.90)
Spinach	< 0.0001	< 0.0001	0.78 (0.70 to 0.87)
Spirits	< 0.0001	< 0.0001	0.81 (0.75 to 0.88)
Tea with sugar	< 0.0001	0.0020	1.23 (1.12 to 1.36)
Tinned tuna	< 0.0001	< 0.0001	0.80 (0.73 to 0.88)
White wine	< 0.0001	0.0279	0.84 (0.77 to 0.92)
Whole milk	< 0.0001	0.0105	1.18 (1.09 to 1.29)
Wholemeal bread	< 0.0001	< 0.0001	0.68 (0.57 to 0.80)

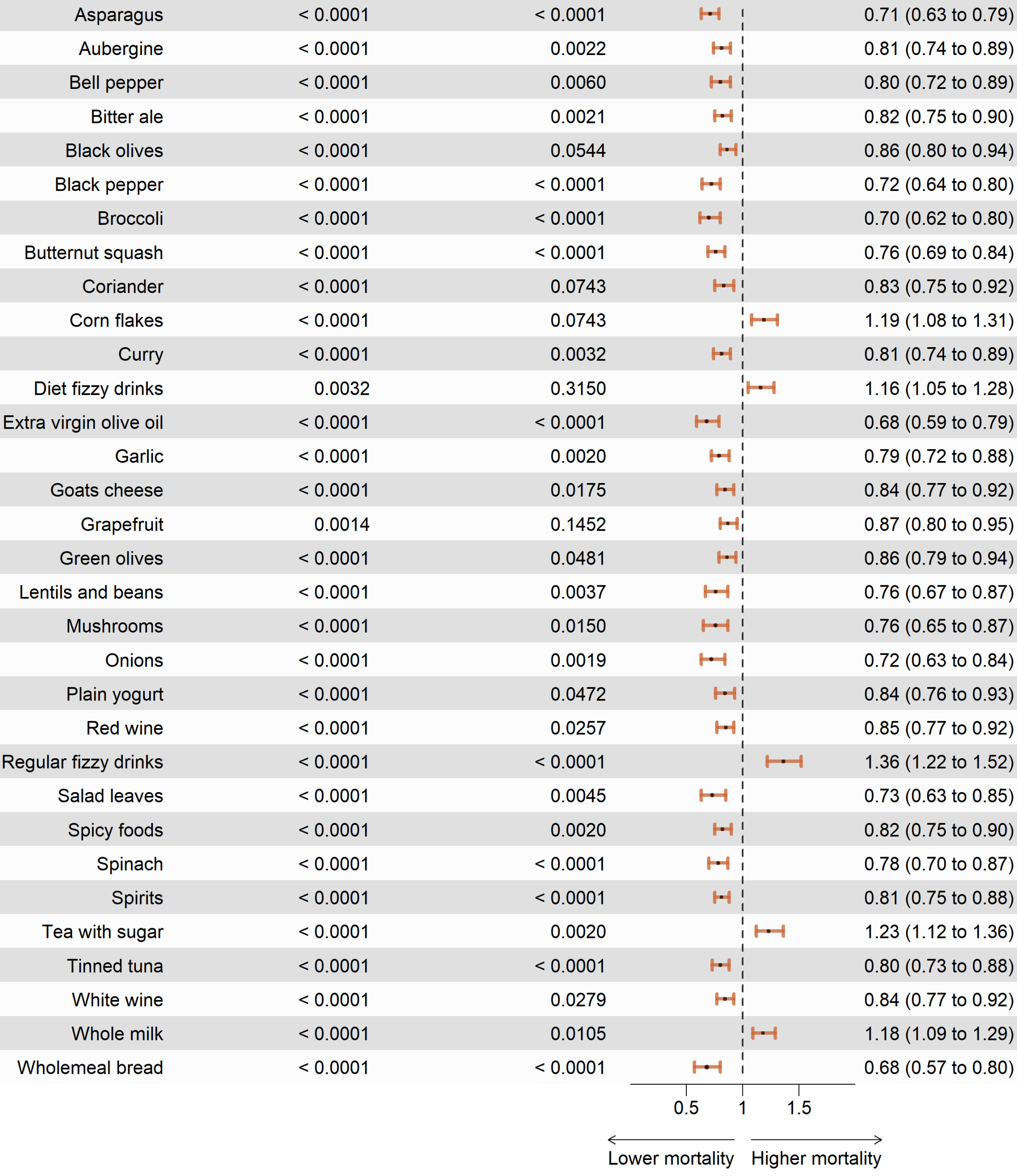

Figure S13. Inclusion of BMI into the model

Name	p-value (unadjusted)	p-value (Holm-adjusted)	HR (95% CI)
Asparagus	< 0.0001	< 0.0001	0.69 (0.62 to 0.77)
Aubergine	< 0.0001	< 0.0001	0.80 (0.73 to 0.88)
Bell pepper	< 0.0001	0.0043	0.80 (0.71 to 0.89)
Bitter ale	< 0.0001	0.0014	0.81 (0.74 to 0.89)
Black olives	< 0.0001	0.0195	0.85 (0.79 to 0.93)
Black pepper	< 0.0001	< 0.0001	0.72 (0.64 to 0.81)
Broccoli	< 0.0001	< 0.0001	0.69 (0.61 to 0.79)
Butternut squash	< 0.0001	< 0.0001	0.75 (0.68 to 0.83)
Coriander	< 0.0001	0.0389	0.82 (0.74 to 0.92)
Corn flakes	< 0.0001	0.0345	1.20 (1.09 to 1.32)
Curry	< 0.0001	0.0043	0.81 (0.74 to 0.90)
Diet fizzy drinks	< 0.0001	0.0043	1.22 (1.11 to 1.35)
Extra virgin olive oil	< 0.0001	< 0.0001	0.67 (0.58 to 0.78)
Garlic	< 0.0001	0.0038	0.80 (0.72 to 0.89)
Goats cheese	< 0.0001	0.0133	0.84 (0.77 to 0.92)
Grapefruit	< 0.0001	0.0512	0.86 (0.79 to 0.93)
Green olives	< 0.0001	0.0182	0.85 (0.79 to 0.93)
Lentils and beans	< 0.0001	0.0023	0.76 (0.67 to 0.86)
Mushrooms	< 0.0001	0.0143	0.76 (0.65 to 0.87)
Onions	< 0.0001	0.0059	0.74 (0.64 to 0.86)
Plain yogurt	< 0.0001	0.0339	0.84 (0.76 to 0.92)
Red wine	< 0.0001	0.0061	0.83 (0.76 to 0.91)
Regular fizzy drinks	< 0.0001	< 0.0001	1.37 (1.23 to 1.53)
Salad leaves	< 0.0001	0.0023	0.72 (0.62 to 0.84)
Spicy foods	< 0.0001	0.0038	0.83 (0.76 to 0.90)
Spinach	< 0.0001	< 0.0001	0.77 (0.69 to 0.85)
Spirits	< 0.0001	< 0.0001	0.82 (0.76 to 0.89)
Tea with sugar	< 0.0001	0.0080	1.21 (1.10 to 1.34)
Tinned tuna	< 0.0001	0.0010	0.80 (0.73 to 0.88)
White wine	< 0.0001	0.0061	0.83 (0.76 to 0.91)
Whole milk	< 0.0001	0.0095	1.19 (1.09 to 1.29)
Wholemeal bread	< 0.0001	< 0.0001	0.67 (0.57 to 0.79)

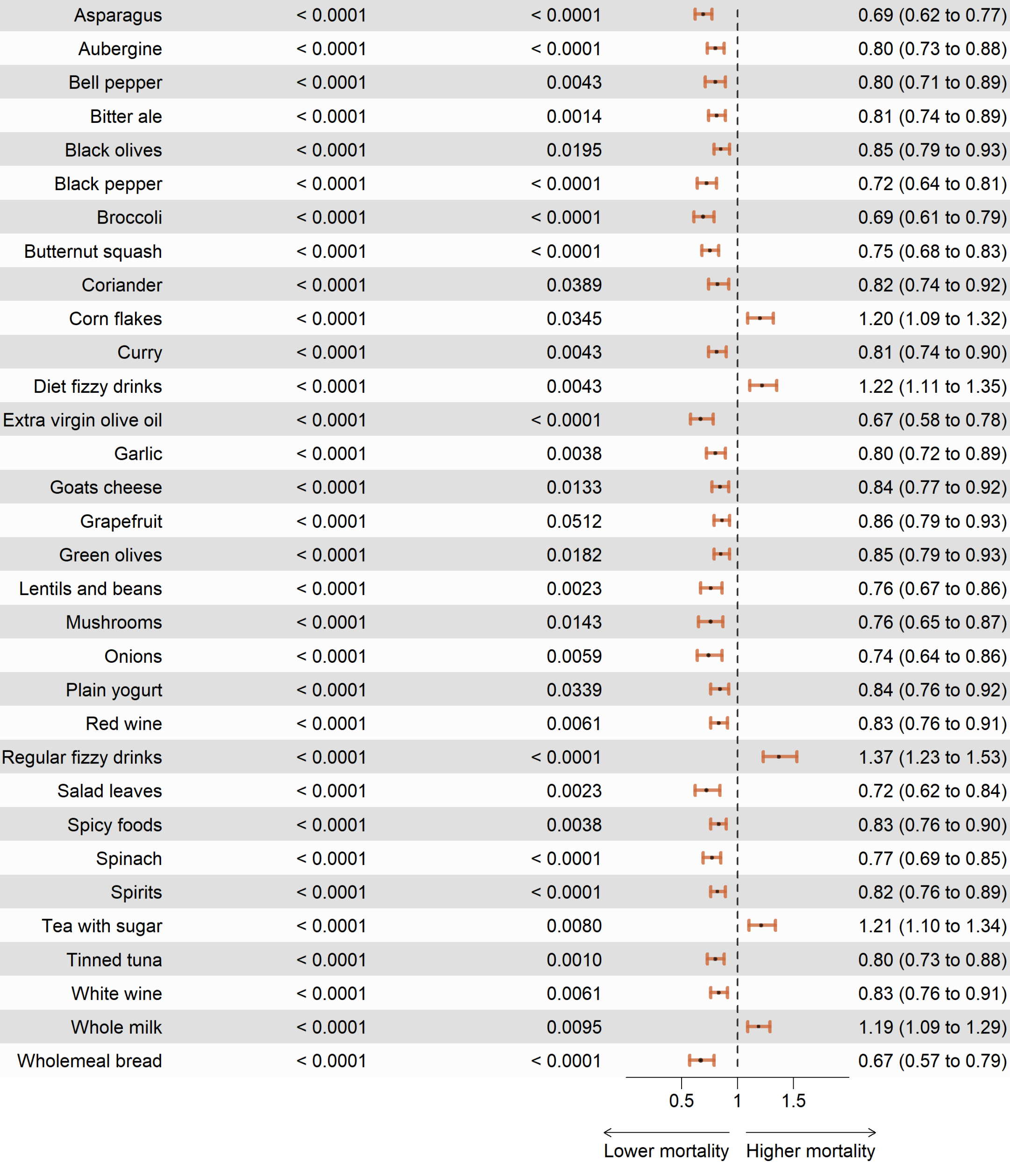

Figure S14. Inclusion of physical activity (MET per week) into the model

Name	p-value (unadjusted)	p-value (Holm-adjusted)	HR (95% CI)
Asparagus	< 0.0001	< 0.0001	0.76 (0.69 to 0.84)
Aubergine	< 0.0001	0.0128	0.85 (0.78 to 0.92)
Bell pepper	< 0.0001	0.0142	0.83 (0.75 to 0.91)
Bitter ale	< 0.0001	0.0034	0.83 (0.77 to 0.91)
Black olives	< 0.0001	0.0909	0.88 (0.81 to 0.95)
Black pepper	< 0.0001	< 0.0001	0.74 (0.67 to 0.82)
Broccoli	< 0.0001	< 0.0001	0.75 (0.67 to 0.84)
Butternut squash	< 0.0001	< 0.0001	0.79 (0.72 to 0.86)
Coriander	< 0.0001	0.0012	0.81 (0.74 to 0.89)
Corn flakes	< 0.0001	0.0236	1.18 (1.08 to 1.28)
Curry	< 0.0001	0.0012	0.82 (0.75 to 0.89)
Diet fizzy drinks	0.0037	0.3656	1.13 (1.04 to 1.23)
Extra virgin olive oil	< 0.0001	< 0.0001	0.72 (0.64 to 0.81)
Garlic	< 0.0001	< 0.0001	0.80 (0.73 to 0.88)
Goats cheese	< 0.0001	0.0074	0.85 (0.78 to 0.92)
Grapefruit	0.0016	0.1587	0.88 (0.82 to 0.95)
Green olives	< 0.0001	0.0873	0.88 (0.81 to 0.95)
Lentils and beans	< 0.0001	0.0194	0.81 (0.73 to 0.91)
Mushrooms	< 0.0001	0.0147	0.78 (0.68 to 0.88)
Onions	< 0.0001	0.0218	0.78 (0.69 to 0.89)
Plain yogurt	0.0089	0.8377	0.89 (0.82 to 0.97)
Red wine	< 0.0001	0.0128	0.85 (0.78 to 0.92)
Regular fizzy drinks	< 0.0001	< 0.0001	1.24 (1.13 to 1.36)
Salad leaves	< 0.0001	0.0054	0.78 (0.69 to 0.88)
Spicy foods	< 0.0001	0.0280	0.86 (0.80 to 0.93)
Spinach	< 0.0001	0.0301	0.84 (0.76 to 0.92)
Spirits	< 0.0001	0.0051	0.85 (0.79 to 0.92)
Tea with sugar	< 0.0001	0.0110	1.20 (1.09 to 1.31)
Tinned tuna	< 0.0001	0.0262	0.85 (0.77 to 0.93)
White wine	0.0010	0.1080	0.87 (0.80 to 0.95)
Whole milk	0.0035	0.3549	1.12 (1.04 to 1.21)
Wholemeal bread	< 0.0001	< 0.0001	0.69 (0.60 to 0.79)

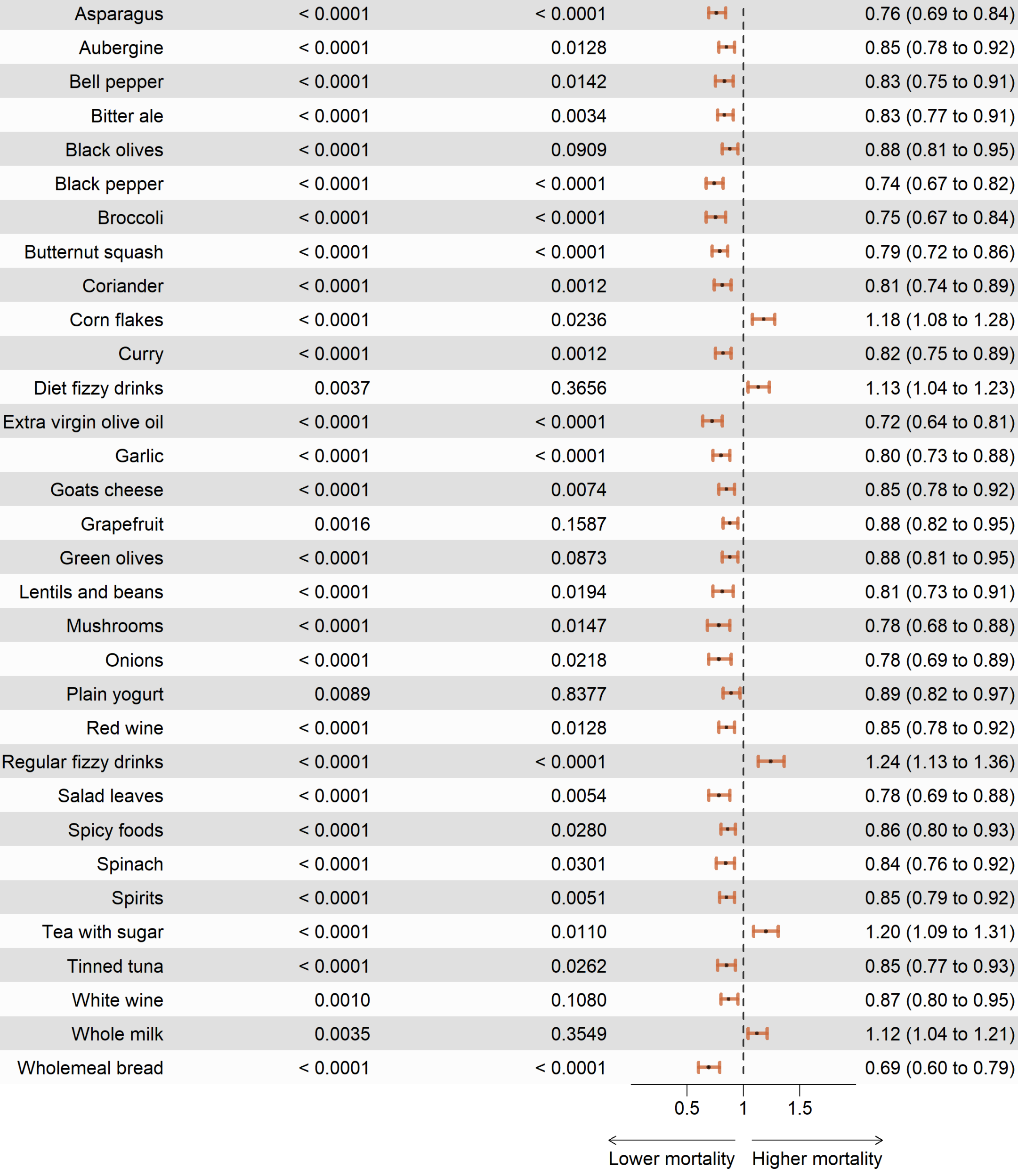

Figure S15. Larger groups by adding level 4 to the low preference group and 6 to the high preference group

Name	p-value (unadjusted)	p-value (Holm-adjusted)	HR (95% CI)
Asparagus	< 0.0001	< 0.0001	0.67 (0.56 to 0.79)
Aubergine	< 0.0001	0.0503	0.77 (0.67 to 0.89)
Bell pepper	0.0062	0.7251	0.80 (0.68 to 0.94)
Bitter ale	0.4062	1.0000	0.93 (0.79 to 1.10)
Black olives	0.0058	0.6945	0.84 (0.75 to 0.95)
Black pepper	< 0.0001	< 0.0001	0.67 (0.57 to 0.79)
Broccoli	0.0026	0.3251	0.69 (0.54 to 0.88)
Butternut squash	< 0.0001	0.0092	0.72 (0.61 to 0.85)
Coriander	0.0646	1.0000	0.86 (0.74 to 1.01)
Corn flakes	< 0.0001	0.1108	1.28 (1.11 to 1.48)
Curry	0.0017	0.2101	0.80 (0.69 to 0.92)
Diet fizzy drinks	0.1827	1.0000	1.11 (0.95 to 1.29)
Extra virgin olive oil	0.0040	0.4932	0.72 (0.57 to 0.90)
Garlic	0.0066	0.7497	0.79 (0.67 to 0.94)
Goats cheese	0.0064	0.7335	0.83 (0.73 to 0.95)
Grapefruit	0.0131	1.0000	0.84 (0.74 to 0.97)
Green olives	0.0062	0.7251	0.84 (0.75 to 0.95)
Lentils and beans	0.0285	1.0000	0.81 (0.67 to 0.98)
Mushrooms	0.0276	1.0000	0.79 (0.64 to 0.97)
Onions	0.0106	1.0000	0.76 (0.61 to 0.94)
Plain yogurt	0.0344	1.0000	0.84 (0.72 to 0.99)
Red wine	0.0049	0.5969	0.84 (0.74 to 0.95)
Regular fizzy drinks	< 0.0001	0.0446	1.43 (1.18 to 1.74)
Salad leaves	0.0081	0.9032	0.70 (0.53 to 0.91)
Spicy foods	0.0012	0.1494	0.81 (0.71 to 0.92)
Spinach	0.0060	0.7058	0.79 (0.66 to 0.93)
Spirits	0.0030	0.3712	0.82 (0.73 to 0.94)
Tea with sugar	< 0.0001	0.0597	1.35 (1.14 to 1.59)
Tinned tuna	0.0819	1.0000	0.87 (0.75 to 1.02)
White wine	< 0.0001	0.0929	0.79 (0.70 to 0.91)
Whole milk	< 0.0001	0.0016	1.34 (1.17 to 1.52)
Wholemeal bread	0.0091	1.0000	0.71 (0.54 to 0.92)

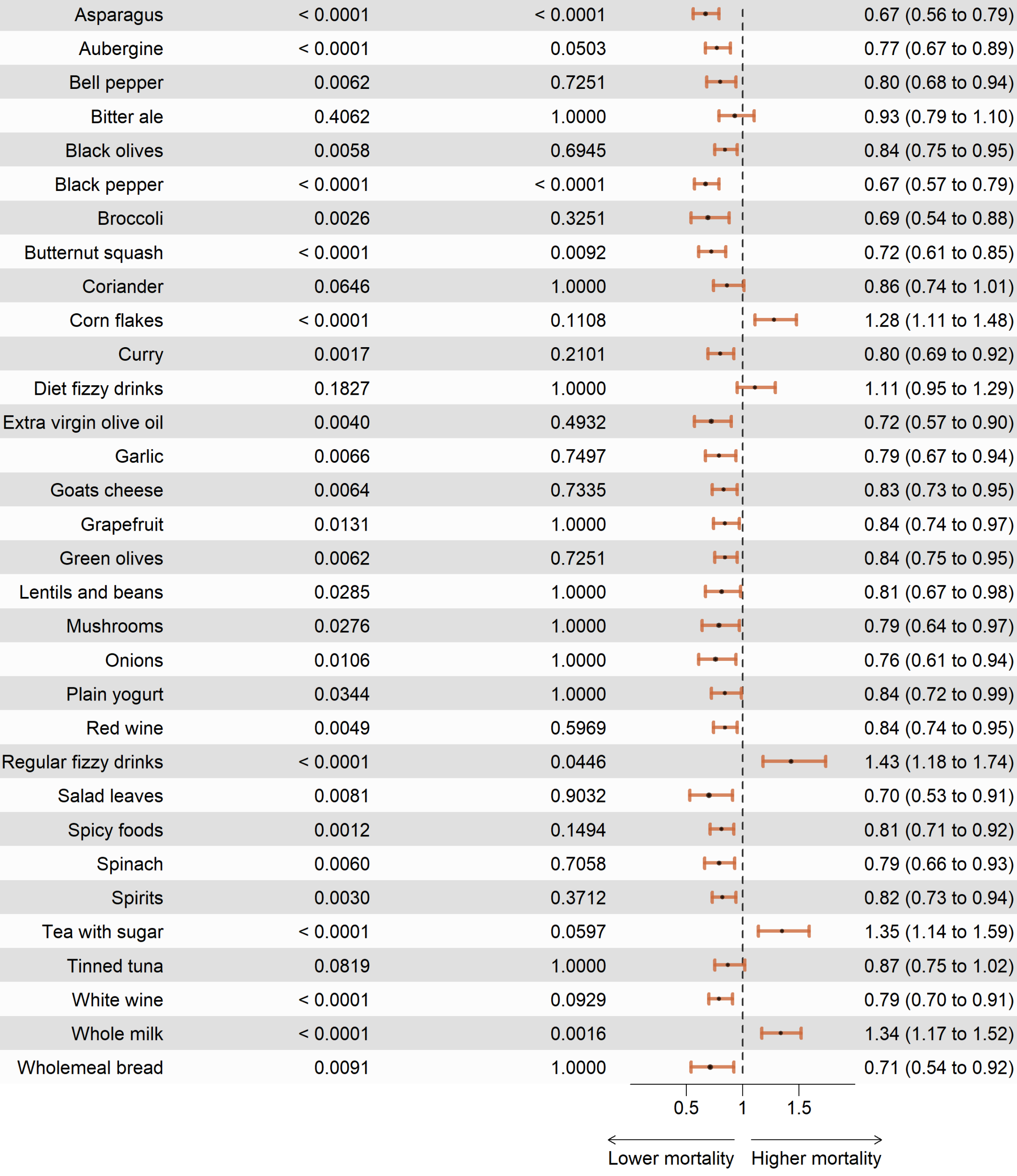

Figure S16. Females only

Name	p-value (unadjusted)	p-value (Holm-adjusted)	HR (95% CI)
Asparagus	< 0.0001	< 0.0001	0.71 (0.62 to 0.82)
Aubergine	0.0018	0.2179	0.82 (0.72 to 0.93)
Bell pepper	0.0021	0.2386	0.79 (0.69 to 0.92)
Bitter ale	< 0.0001	< 0.0001	0.77 (0.69 to 0.86)
Black olives	0.0107	1.0000	0.86 (0.77 to 0.97)
Black pepper	< 0.0001	0.0895	0.76 (0.65 to 0.89)
Broccoli	< 0.0001	0.0010	0.70 (0.60 to 0.82)
Butternut squash	< 0.0001	0.0098	0.77 (0.68 to 0.88)
Coriander	0.0012	0.1456	0.78 (0.68 to 0.91)
Corn flakes	0.0727	1.0000	1.13 (0.99 to 1.30)
Curry	0.0058	0.6129	0.83 (0.72 to 0.95)
Diet fizzy drinks	< 0.0001	0.0024	1.31 (1.16 to 1.48)
Extra virgin olive oil	< 0.0001	< 0.0001	0.65 (0.54 to 0.78)
Garlic	0.0017	0.2015	0.81 (0.71 to 0.92)
Goats cheese	0.0051	0.5477	0.84 (0.75 to 0.95)
Grapefruit	0.0145	1.0000	0.87 (0.77 to 0.97)
Green olives	0.0087	0.8805	0.86 (0.77 to 0.96)
Lentils and beans	< 0.0001	0.0106	0.71 (0.60 to 0.84)
Mushrooms	< 0.0001	0.1074	0.72 (0.59 to 0.87)
Onions	< 0.0001	0.1073	0.71 (0.58 to 0.87)
Plain yogurt	0.0039	0.4253	0.83 (0.74 to 0.94)
Red wine	0.0063	0.6488	0.84 (0.74 to 0.95)
Regular fizzy drinks	< 0.0001	0.0027	1.34 (1.17 to 1.53)
Salad leaves	< 0.0001	0.0758	0.73 (0.61 to 0.87)
Spicy foods	0.0061	0.6399	0.84 (0.74 to 0.95)
Spinach	< 0.0001	0.0045	0.75 (0.65 to 0.86)
Spirits	< 0.0001	0.0338	0.82 (0.73 to 0.91)
Tea with sugar	0.0143	1.0000	1.16 (1.03 to 1.30)
Tinned tuna	< 0.0001	0.0013	0.75 (0.67 to 0.85)
White wine	0.0186	1.0000	0.86 (0.76 to 0.98)
Whole milk	0.1956	1.0000	1.08 (0.96 to 1.20)
Wholemeal bread	< 0.0001	0.0126	0.65 (0.52 to 0.80)

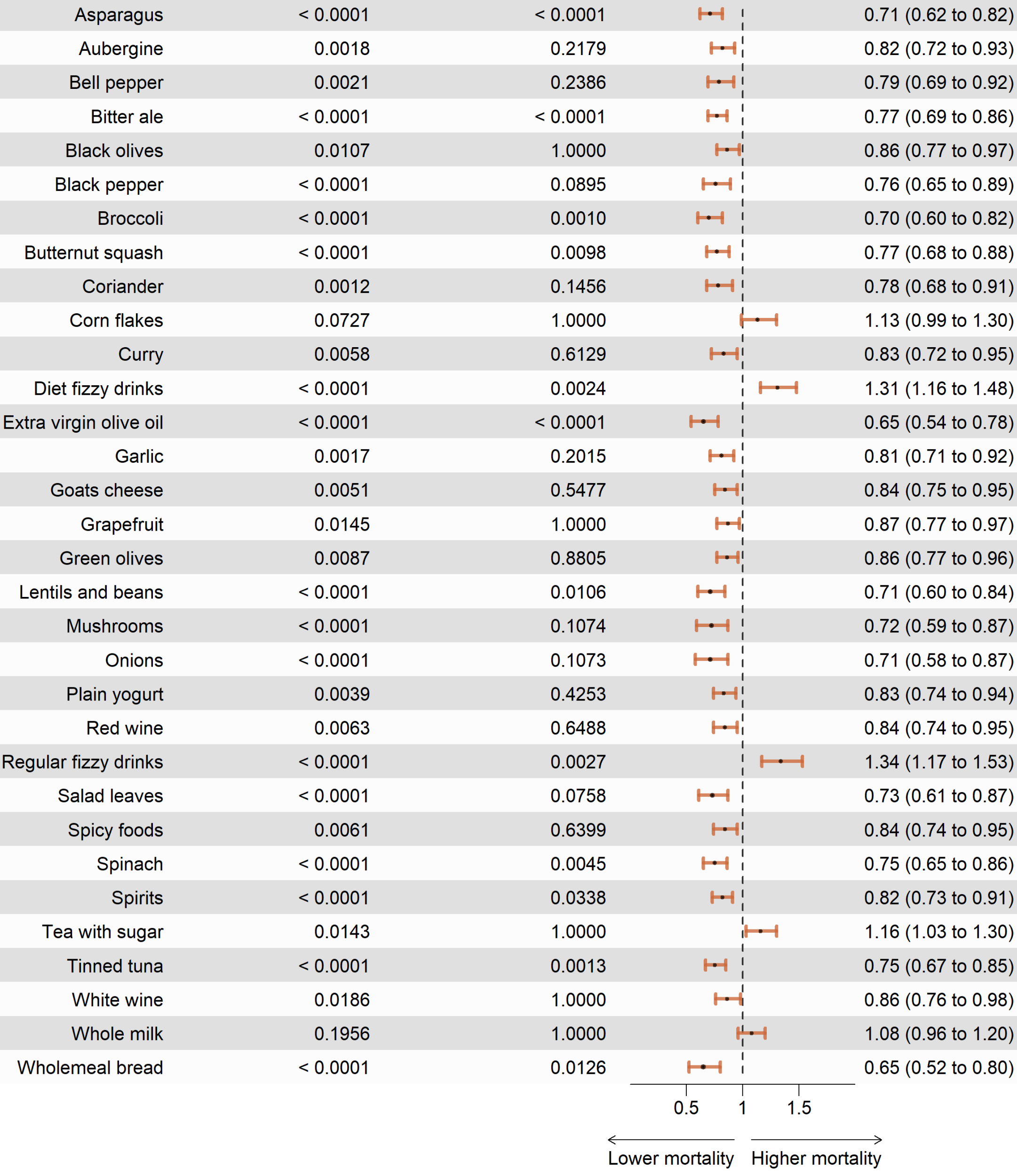

Figure S17. Males only

Name	p-value (unadjusted)	p-value (Holm-adjusted)	HR (95% CI)
Asparagus	< 0.0001	< 0.0001	0.63 (0.54 to 0.74)
Aubergine	0.0070	0.7506	0.82 (0.71 to 0.95)
Bell pepper	< 0.0001	0.0198	0.74 (0.63 to 0.86)
Bitter ale	0.0226	1.0000	0.85 (0.74 to 0.98)
Black olives	0.0211	1.0000	0.87 (0.77 to 0.98)
Black pepper	< 0.0001	< 0.0001	0.67 (0.57 to 0.79)
Broccoli	0.0022	0.2465	0.72 (0.58 to 0.89)
Butternut squash	< 0.0001	0.0065	0.73 (0.63 to 0.85)
Coriander	0.0067	0.7263	0.80 (0.68 to 0.94)
Corn flakes	< 0.0001	0.0709	1.30 (1.12 to 1.51)
Curry	0.0014	0.1574	0.80 (0.69 to 0.91)
Diet fizzy drinks	< 0.0001	0.0329	1.31 (1.13 to 1.52)
Extra virgin olive oil	< 0.0001	< 0.0001	0.56 (0.46 to 0.68)
Garlic	< 0.0001	< 0.0001	0.71 (0.61 to 0.82)
Goats cheese	0.0018	0.2047	0.81 (0.71 to 0.92)
Grapefruit	0.0467	1.0000	0.87 (0.77 to 1.00)
Green olives	0.0032	0.3473	0.83 (0.74 to 0.94)
Lentils and beans	< 0.0001	0.0925	0.73 (0.60 to 0.88)
Mushrooms	0.0748	1.0000	0.82 (0.66 to 1.02)
Onions	< 0.0001	0.0031	0.66 (0.54 to 0.80)
Plain yogurt	< 0.0001	0.0211	0.75 (0.65 to 0.87)
Red wine	< 0.0001	0.0887	0.80 (0.70 to 0.91)
Regular fizzy drinks	< 0.0001	< 0.0001	1.50 (1.27 to 1.76)
Salad leaves	0.0031	0.3454	0.71 (0.56 to 0.89)
Spicy foods	< 0.0001	0.0105	0.77 (0.68 to 0.88)
Spinach	< 0.0001	0.0065	0.72 (0.61 to 0.84)
Spirits	< 0.0001	0.0198	0.79 (0.70 to 0.89)
Tea with sugar	< 0.0001	0.0364	1.32 (1.14 to 1.54)
Tinned tuna	0.0514	1.0000	0.86 (0.74 to 1.00)
White wine	< 0.0001	0.0033	0.75 (0.65 to 0.86)
Whole milk	0.0215	1.0000	1.16 (1.02 to 1.32)
Wholemeal bread	< 0.0001	0.0700	0.63 (0.48 to 0.82)

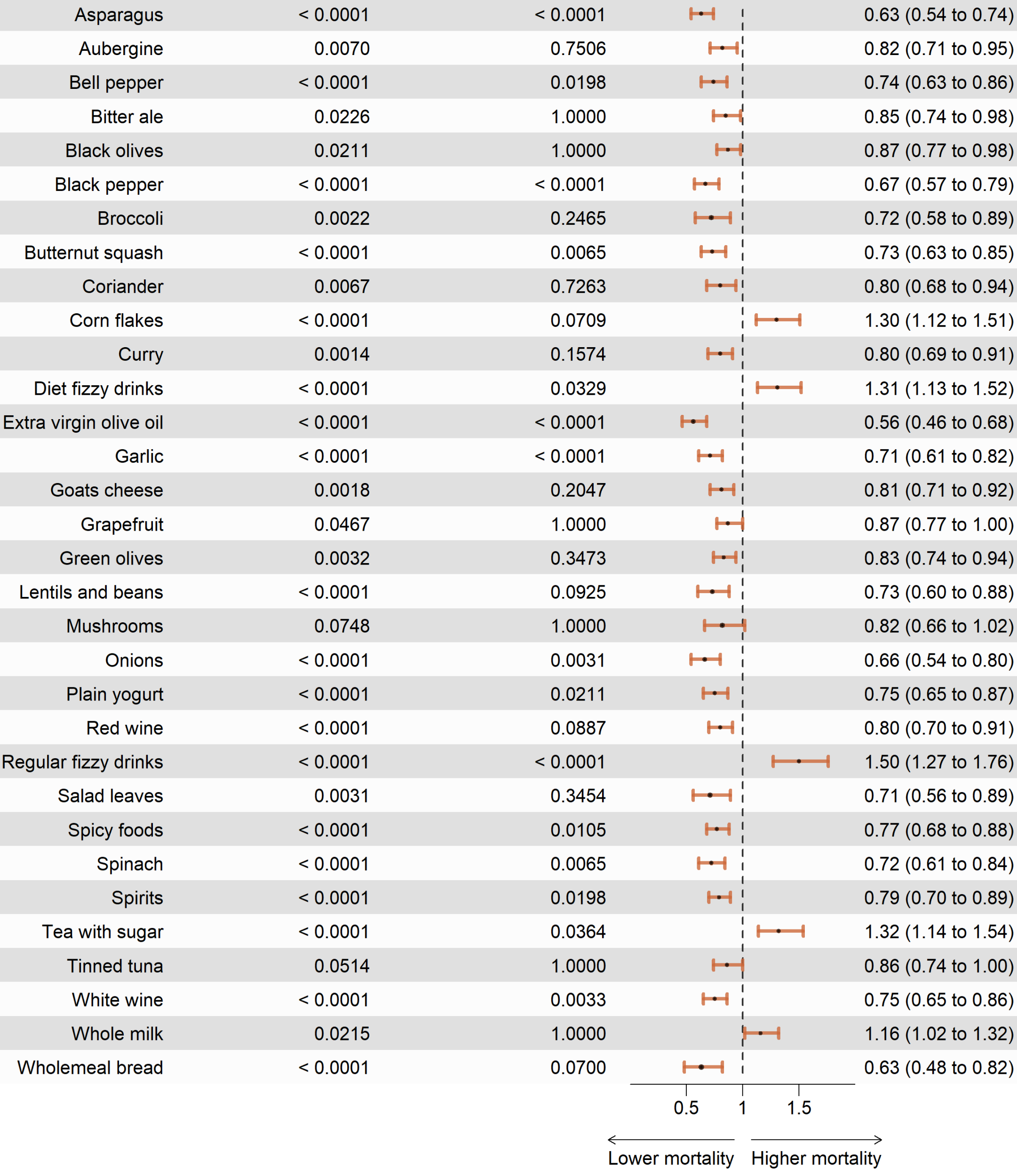

Figure S18. Non-smokers only

Name	p-value (unadjusted)	p-value (Holm-adjusted)	HR (95% CI)
Asparagus	< 0.0001	0.0335	0.73 (0.62 to 0.86)
Aubergine	< 0.0001	0.0253	0.77 (0.67 to 0.88)
Bell pepper	0.0027	0.3603	0.78 (0.66 to 0.92)
Bitter ale	< 0.0001	0.0066	0.75 (0.66 to 0.86)
Black olives	0.0125	1.0000	0.85 (0.75 to 0.97)
Black pepper	0.0260	1.0000	0.80 (0.66 to 0.97)
Broccoli	< 0.0001	0.0049	0.67 (0.55 to 0.81)
Butternut squash	0.0058	0.7426	0.81 (0.70 to 0.94)
Coriander	0.0320	1.0000	0.84 (0.71 to 0.98)
Corn flakes	0.2397	1.0000	1.09 (0.94 to 1.26)
Curry	0.0167	1.0000	0.83 (0.71 to 0.97)
Diet fizzy drinks	0.1271	1.0000	1.12 (0.97 to 1.29)
Extra virgin olive oil	0.0393	1.0000	0.78 (0.61 to 0.99)
Garlic	0.1345	1.0000	0.88 (0.74 to 1.04)
Goats cheese	0.0745	1.0000	0.88 (0.77 to 1.01)
Grapefruit	0.0273	1.0000	0.86 (0.76 to 0.98)
Green olives	0.0171	1.0000	0.86 (0.76 to 0.97)
Lentils and beans	0.0030	0.3929	0.74 (0.61 to 0.90)
Mushrooms	0.0037	0.4830	0.72 (0.57 to 0.90)
Onions	0.1275	1.0000	0.83 (0.65 to 1.06)
Plain yogurt	0.6962	1.0000	0.97 (0.84 to 1.13)
Red wine	0.0988	1.0000	0.89 (0.77 to 1.02)
Regular fizzy drinks	0.0034	0.4478	1.29 (1.09 to 1.53)
Salad leaves	0.0083	1.0000	0.73 (0.58 to 0.92)
Spicy foods	0.0307	1.0000	0.86 (0.75 to 0.99)
Spinach	0.0064	0.8113	0.80 (0.68 to 0.94)
Spirits	0.0042	0.5372	0.83 (0.73 to 0.94)
Tea with sugar	0.1604	1.0000	1.11 (0.96 to 1.28)
Tinned tuna	< 0.0001	0.0105	0.75 (0.65 to 0.86)
White wine	0.0365	1.0000	0.86 (0.75 to 0.99)
Whole milk	0.0041	0.5297	1.21 (1.06 to 1.37)
Wholemeal bread	0.0339	1.0000	0.75 (0.58 to 0.98)

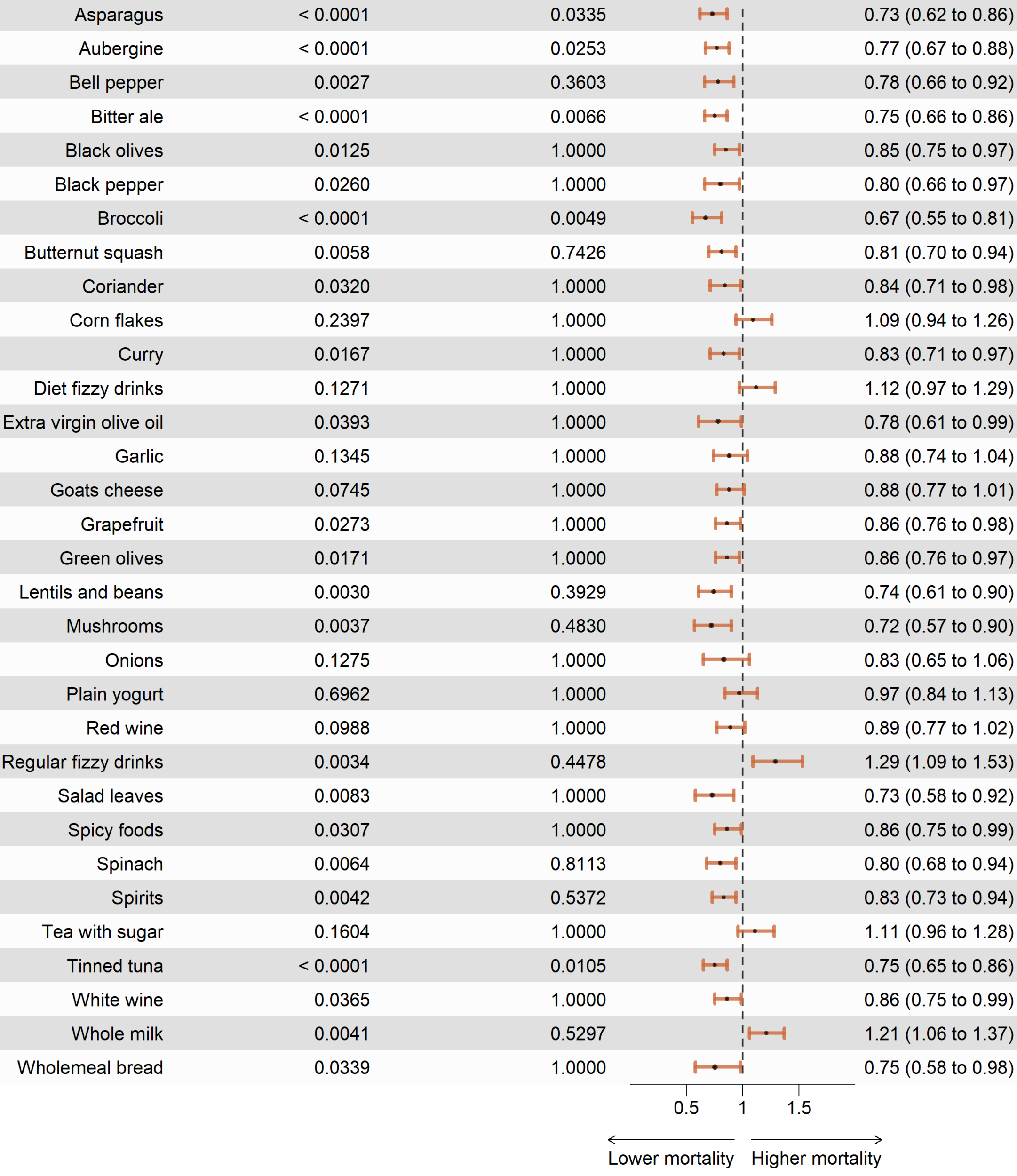

Figure S19. Previous smokers only

Name	p-value (unadjusted)	p-value (Holm-adjusted)	HR (95% CI)
Asparagus	0.0484	1.0000	0.73 (0.53 to 1.00)
Aubergine	0.0475	1.0000	0.75 (0.57 to 1.00)
Bell pepper	0.6137	1.0000	1.09 (0.78 to 1.54)
Bitter ale	0.1120	1.0000	0.80 (0.61 to 1.05)
Black olives	0.0118	1.0000	0.73 (0.58 to 0.93)
Black pepper	0.0073	0.9519	0.63 (0.45 to 0.88)
Broccoli	0.0067	0.8848	0.63 (0.45 to 0.88)
Butternut squash	< 0.0001	0.0051	0.54 (0.40 to 0.72)
Coriander	0.1188	1.0000	0.78 (0.57 to 1.07)
Corn flakes	0.1246	1.0000	1.27 (0.94 to 1.73)
Curry	0.0505	1.0000	0.75 (0.56 to 1.00)
Diet fizzy drinks	0.0371	1.0000	1.34 (1.02 to 1.76)
Extra virgin olive oil	0.4166	1.0000	0.83 (0.53 to 1.30)
Garlic	0.7797	1.0000	0.95 (0.67 to 1.34)
Goats cheese	0.0253	1.0000	0.74 (0.57 to 0.96)
Grapefruit	0.0202	1.0000	0.74 (0.57 to 0.95)
Green olives	0.0768	1.0000	0.80 (0.63 to 1.02)
Lentils and beans	0.3619	1.0000	0.84 (0.58 to 1.22)
Mushrooms	0.0058	0.7719	0.59 (0.41 to 0.86)
Onions	0.5134	1.0000	0.85 (0.52 to 1.39)
Plain yogurt	< 0.0001	0.0739	0.63 (0.48 to 0.82)
Red wine	< 0.0001	0.1067	0.65 (0.50 to 0.83)
Regular fizzy drinks	0.0195	1.0000	1.44 (1.06 to 1.96)
Salad leaves	0.0158	1.0000	0.62 (0.42 to 0.91)
Spicy foods	0.4598	1.0000	0.90 (0.69 to 1.18)
Spinach	0.0534	1.0000	0.74 (0.55 to 1.00)
Spirits	0.3123	1.0000	0.88 (0.68 to 1.13)
Tea with sugar	0.0041	0.5532	1.41 (1.12 to 1.79)
Tinned tuna	0.0239	1.0000	0.73 (0.56 to 0.96)
White wine	0.4767	1.0000	0.91 (0.70 to 1.18)
Whole milk	0.0699	1.0000	1.26 (0.98 to 1.61)
Wholemeal bread	0.0043	0.5817	0.57 (0.39 to 0.84)

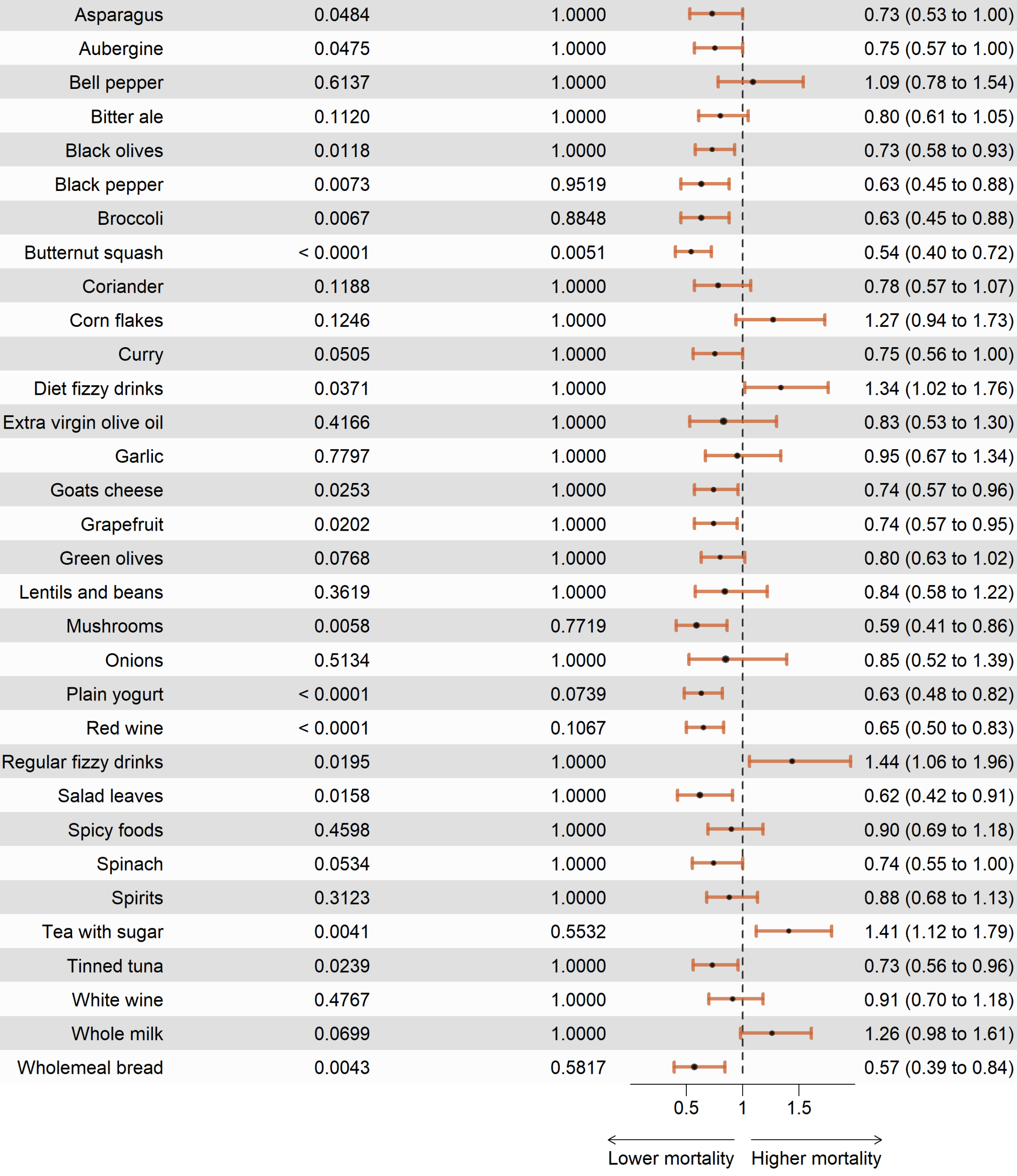

Figure S20. Current smokers only

**Exluded (not a food preference)**
Bicycling
Cigarette smoking
Exercising alone
Exercising with others
Going to a cafe
Going to the gym
Going to the pub
Taking the stairs
Watching television
Working up a sweat

Table S1. All questionnaire items excluded from the analysis due to not being food preferences

Food preference	n	Food preference	n	Food preference	n	Food preference	n
Adding salt to foods	176,888	Cheesecake	176,643	Honey	176,497	Salad dressing	176,722
Aniseed	168,496	Cherries	176,795	Horseradish/wasabi	169,424	Salad leaves	176,928
Apple juice	176,631	Chicken	175,737	Ice cream	176,941	Salami	171,226
Apples	176,962	Chilli pepper	174,765	Jam	177,025	Salmon	175,862
Asparagus	172,876	Chips/French fries	177,025	Kiwi fruit	174,484	Salty foods	177,025
Aubergine	169,173	Cod	176,004	Lager	173,660	Salty pretzels	167,270
Avocados	170,103	Coffee with sugar	175,458	Lamb	175,583	Sardines	174,369
Bacon	175,247	Coffee without sugar	175,485	Lemons	176,901	Sausages (meat)	175,636
Baked/steamed fish	175,477	Coriander	171,349	Lentils/beans	176,531	Savoury biscuits	176,682
Bananas	176,920	Corn flakes	176,777	Liver	175,359	Shellfish	172,053
Barbequed/grilled meat	175,520	Cream	176,884	Mackerel	173,641	Skimmed milk	175,948
Beef steak	175,420	Croissant	175,976	Marzipan	176,409	Smoked fish	175,256
Beetroot	176,713	Cucumber	176,954	Mayonnaise	176,786	Soft cheese	176,648
Bell pepper	158,248	Curry	176,690	Melon	176,886	Soy sauce	174,472
Biscuits	176,980	Dairy products	176,798	Milk chocolate	176,860	Soya milk	126,901
Bitter foods	174,691	Dark chocolate	176,907	Mushrooms	176,887	Spicy foods	176,952
Bitter/ale	171,489	Diet fizzy drinks	174,208	Onions	176,969	Spinach	175,323
Black olives	173,366	Dried fruit	176,674	Orange juice	176,910	Spirits	174,666
Black pepper	176,171	Eggs	176,838	Oranges	176,907	Strawberries	176,889
Blue cheese	174,319	Extra virgin olive oil	174,592	Pasta	176,841	Sweet coffee house drinks	165,225
Bolognese sauce	175,133	Fatty foods	176,915	Pears	176,914	Sweet foods	176,982
Broad beans	176,289	Fresh tomatoes	176,861	Pizza	176,777	Tea with sugar	175,422
Broccoli	176,834	Fried chicken	174,234	Plain yogurt	176,064	Tea without sugar	175,354
Brown rice	173,983	Fried/battered fish	176,071	Plums	176,796	Tomato ketchup	176,822
Brussel sprouts	176,883	Fruit	176,869	Pollock	144,941	Tinned tuna	175,394
Burgers (meat)	175,529	Garlic	176,823	Pork chop	175,012	Turnip (white)	172,120
Burn of spicy foods	170,912	Gherkins	172,937	Porridge	176,511	Vegetables	176,918
Butter on bread	176,790	Globe artichoke	129,121	Potato crisps	177,009	Vinegar	176,999
Butternut sqash	165,397	Goats cheese	167,313	Potatoes	177,034	Whisky	173,649
Cabbage	176,963	Grapefruit	174,969	Prawns	174,941	White bread	176,942
Cake	176,970	Green olives	173,922	Raw carrots	176,722	White rice	176,947
Cake icing	176,895	Haddock	175,475	Red meat	175,753	White wine	175,537
Capers	156,342	Ham	175,219	Red wine	175,421	Wholegrain breakfast cereal	174,878
Cauliflower	176,930	Hard cheese	176,719	Regular fizzy drinks	176,175	Whole milk	176,558
Cereal/granola bar	172,068	Herring	166,867	Roast chicken	175,729	Wholemeal bread	176,776

Table S2. Food preference items included in analysis and number of participants included in the respective models (n)

	Main analysis		Sensitivity analyses										
Name	p-values unadjusted	p-values Holm adjusted	S5 p-values Holm adjusted	S6 p-values Holm adjusted	S7 p-values Holm adjusted	S8 p-values Holm adjusted	S9 p-values Holm adjusted	S10 p-values Holm adjusted	S11 p-values Holm adjusted	S12 p-values Holm adjusted	S13 p-values Holm adjusted	S14 p-values Holm adjusted	S15 p-values Holm adjusted
Adding salt to foods	0.193728237	1	1	1	1	1	1	1	1	1	1	1	1
Aniseed	0.262991888	1	1	1	1	1	1	1	1	1	1	1	1
Apple juice	0.019232403	1	0.377840456	1	1	1	0.515595	1	1	0.955142	1	1	1
Apples	0.089521479	1	1	1	1	1	1	1	1	1	1	0.893048	1
Asparagus	3.84226E-11	5.38E-09	1.69832E-07	1.01811E-07	5.99E-08	1.32E-06	1.48E-06	8.95E-06	2.41E-06	0.000158	1.01E-06	5.78E-06	7E-09
Aubergine	1.80875E-06	0.000241	0.022150917	0.000104447	0.002219	0.00272	0.017717	0.010444	0.006322	0.007276	0.011115	0.020932	0.000348
Avocados	0.002546043	0.254604	1	0.359556547	0.961844	0.234577	1	1	1	1	1	0.558612	0.28622
Bacon	0.131451449	1	1	1	1	1	1	1	1	1	1	1	1
Baked steamed fish	0.150513468	1	1	1	1	1	1	1	1	1	1	1	1
Bananas	0.001157479	0.121535	0.241439224	0.04379857	0.125776	1	0.901276	0.153194	0.163135	1	1	0.056985	0.137885
Barbequed grilled meat	0.019892133	1	1	1	0.283415	0.418168	1	0.348149	0.205059	0.408626	1	1	1
Beef steak	0.923387839	1	1	1	1	1	1	1	1	1	1	1	1
Beetroot	0.001054829	0.111812	1	0.621754623	0.16592	0.346533	1	0.576673	0.051534	0.14309	0.308981	0.558612	0.151237
Bell pepper	2.95597E-05	0.003695	0.003200691	0.300885697	0.006042	0.009244	0.050067	0.006781	0.00889	0.121155	0.02405	0.623673	0.004264
Biscuits	0.677245518	1	1	1	1	1	1	1	1	1	1	1	1
Bitter foods	0.007812754	0.726586	1	1	0.683079	1	1	1	1	1	0.718162	1	0.883046
Bitter ale	9.45493E-06	0.00122	0.032646106	0.039977855	0.002142	0.002393	0.013775	0.017871	0.002159	0.028929	0.032481	0.499035	0.001383
Black olives	0.000124679	0.014089	0.477241307	0.386702597	0.054356	0.036987	0.320893	0.135233	0.173864	0.175285	0.138409	0.000902	0.019472
Black pepper	1.59337E-08	2.18E-06	0.000125134	8.8538E-05	1.62E-06	1.42E-06	1.04E-05	1.11E-05	6.02E-06	0.001752	3.85E-06	4.83E-05	2.37E-06
Blue cheese	0.340624091	1	1	1	1	1	1	1	1	1	1	1	1
Bolognese sauce	0.193710698	1	1	1	1	1	1	1	1	1	1	1	1
Broad beans	0.001632787	0.168177	0.27545325	0.579356696	0.341076	1	1	0.723375	0.416789	1	0.547783	0.637093	0.225607
Broccoli	2.50279E-08	3.4E-06	0.019280017	0.001042158	2.12E-05	2.94E-05	0.000378	9.76E-05	0.000278	0.000338	0.004046	0.010659	5.63E-06
Brown rice	0.000672209	0.072599	0.410629185	0.5423675	0.148809	0.007123	0.220036	0.30242	0.063987	1	0.438949	1	0.088022
Brussel sprouts	0.035813173	1	1	1	1	1	1	1	1	1	1	0.426234	1
Burgers (meat)	0.312284974	1	1	1	1	1	1	1	1	1	1	1	1
Burn of spicy foods	0.049145375	1	1	1	1	1	1	1	1	1	1	1	1
Butter on bread	0.440665235	1	1	1	1	1	1	1	1	1	1	1	1

Table S3. Exact p-values of the main analysis and all sensitivity analyses

	Main analysis		Sensitivity analyses										
Name	p-values unadjusted	p-values Holm adjusted	S5 p-values Holm adjusted	S6 p-values Holm adjusted	S7 p-values Holm adjusted	S8 p-values Holm adjusted	S9 p-values Holm adjusted	S10 p-values Holm adjusted	S11 p-values Holm adjusted	S12 p-values Holm adjusted	S13 p-values Holm adjusted	S14 p-values Holm adjusted	S15 p-values Holm adjusted
Butternut squash	1.47618E-08	2.04E-06	0.000131	0.000129276	1.43E-05	3.38E-06	0.000124	1.52E-05	0.000118	0.000206	7.95E-05	8.28E-05	2.97E-06
Cabbage	0.088528329	1	1	1	1	1	1	1	1	1	1	1	1
Cake	0.324786431	1	1	1	1	1	1	1	1	1	1	1	1
Cake icing	0.824209808	1	1	1	1	1	1	1	1	1	1	1	1
Capers	0.007163292	0.673349	1	0.295768717	0.937461	1	1	0.912088	1	1	0.638006	1	0.765279
Cauliflower	0.448476914	1	1	1	1	1	1	1	1	1	1	1	1
Cereal granola bar	0.152468475	1	1	1	1	1	1	1	1	1	1	1	1
Cheesecake	0.344298874	1	1	1	1	1	1	1	1	1	1	1	1
Cherries	0.15873099	1	1	1	1	1	1	1	1	1	1	1	1
Chicken	0.891268562	1	1	1	1	1	1	1	1	1	1	1	1
Chilli pepper	0.001203288	0.125142	1	1	0.09418	0.029344	0.462499	0.095198	0.160457	0.783772	0.272302	0.81679	0.145768
Chips French fries	0.200345215	1	1	1	1	1	1	1	1	1	1	1	1
Cod	0.183139455	1	1	1	1	1	1	1	1	1	1	1	1
Coffee with sugar	0.021890937	1	1	1	1	1	0.830343	1	0.644777	0.936488	0.642223	1	1
Coffee without sugar	0.005542804	0.537652	1	0.555550376	0.288925	0.243357	0.281882	1	0.416789	0.42332	1	1	0.567415
Coriander	0.000284856	0.031334	0.053831948	0.20327436	0.074301	0.000477	0.262442	0.282927	0.260192	0.626453	0.297062	0.133567	0.03889
Corn flakes	0.000234432	0.026022	0.047605802	1	0.074272	0.010915	0.126107	0.096273	0.018005	0.064161	0.038919	0.485174	0.034506
Cream	0.360384068	1	1	1	1	1	1	1	1	1	1	1	1
Croissant	0.022942406	1	1	0.418606831	1	0.529283	1	1	1	1	1	1	1
Cucumber	0.540980178	1	1	1	1	1	1	1	1	1	1	1	1
Curry	3.60566E-05	0.004399	0.057450627	0.026126156	0.00317	0.001568	0.002926	0.002522	0.007831	0.013668	0.034462	0.557768	0.004279
Dairy products	0.490423884	1	1	1	1	1	1	1	1	1	1	1	1
Dark chocolate	0.29716973	1	1	1	1	1	1	1	1	1	1	1	1
Diet fizzy drinks	2.15027E-05	0.002709	0.130884719	0.19479126	0.315013	0.007975	1	1	0.133369	0.331968	0.605148	1	0.004279
Dried fruit	0.018938255	1	1	1	1	1	1	1	1	1	1	1	1
Eggs	0.071181788	1	1	1	1	1	1	1	1	1	1	1	1
Extra virgin olive oil	5.31065E-08	7.17E-06	0.000533669	0.000652011	2.55E-05	6E-06	0.002039	0.000111	7.42E-06	0.006084	0.000576	0.000121	7.89E-06
Fatty foods	0.024472666	1	1	1	0.209225	1	0.567576	0.141535	0.519367	0.584282	1	0.769051	1
Fresh tomatoes	0.765519411	1	1	1	1	1	1	1	1	1	1	1	1
Fried chicken	0.643669093	1	1	1	1	1	1	1	1	1	1	1	1

Table S3. Exact p-values of the main analysis and all sensitivity analyses (continued)

	Main analysis		Sensitivity analyses										
Name	p-values unadjusted	p-values Holm adjusted	S5 p-values Holm adjusted	S6 p-values Holm adjusted	S7 p-values Holm adjusted	S8 p-values Holm adjusted	S9 p-values Holm adjusted	S10 p-values Holm adjusted	S11 p-values Holm adjusted	S12 p-values Holm adjusted	S13 p-values Holm adjusted	S14 p-values Holm adjusted	S15 p-values Holm adjusted
Fried battered fish	0.231226494	1	1	1	1	1	1	1	1	1	1	1	1
Fruit	0.741987911	1	1	1	1	1	1	1	1	1	1	1	1
Garlic	3.06105E-05	0.003791	1	0.2240222	0.001962	0.000862	0.048772	0.006825	0.004404	0.094346	0.007151	0.207291	0.003841
Gherkins	0.231885394	1	1	1	1	1	1	1	1	1	1	1	1
Globe artichoke	0.098775657	1	1	1	1	1	1	1	1	1	1	1	1
Goats cheese	0.00010319	0.01197	0.075662457	0.026126156	0.017515	0.006582	0.046796	0.035533	0.026096	0.077578	0.051397	0.479979	0.013295
Grapefruit	0.000434883	0.047402	0.094765305	0.24511439	0.145247	0.061266	0.497344	0.051335	0.227243	0.108008	0.246836	0.442881	0.051163
Green olives	0.000119998	0.01368	0.413914868	0.197753641	0.048112	0.036852	0.106575	0.066626	0.18131	0.510934	0.072526	0.002811	0.018222
Haddock	0.598186641	1	1	1	1	1	1	1	1	1	1	1	1
Ham	0.007818305	0.726586	0.456733238	0.423831088	1	1	1	1	1	1	0.902816	1	0.95619
Hard cheese	0.354629341	1	1	1	1	1	1	1	1	1	1	1	1
Herring	0.546860926	1	1	1	1	1	1	1	1	1	1	1	1
Honey	0.233019926	1	1	1	1	1	1	1	1	1	1	1	1
Horseradish wasabi	0.230180563	1	1	1	1	1	1	1	1	1	1	1	1
Ice cream	0.353320367	1	1	1	1	1	1	1	1	1	1	1	1
Jam	0.992655121	1	1	1	1	1	1	1	1	1	1	1	1
Kiwi fruit	0.017057457	1	1	1	1	1	1	1	1	1	1	0.305957	1
Lager	0.009805461	0.882492	1	1	1	1	1	1	1	1	1	1	0.883046
Lamb	0.315777904	1	1	1	1	1	1	1	1	1	1	1	1
Lemons	0.013902631	1	1	1	1	0.061883	1	1	1	1	1	0.151587	1
Lentils and beans	1.20968E-05	0.001548	0.001398484	0.00306507	0.00374	0.010385	0.040762	0.052375	0.009795	0.024403	0.135814	0.009477	0.002299
Liver	0.093223426	1	1	1	1	1	1	1	1	1	1	1	1
Mackerel	0.011974523	1	1	1	1	1	1	1	0.867019	1	1	1	1
Marzipan	0.540465261	1	1	1	1	1	1	1	1	1	1	1	1
Mayonnaise	0.955854987	1	1	1	1	1	1	1	1	1	1	1	1
Melon	0.299438388	1	1	1	1	1	1	1	1	1	1	1	1
Milk chocolate	0.019259567	1	1	1	0.724853	1	1	0.817768	0.916667	1	1	1	1
Mushrooms	0.00010906	0.012542	0.060282173	0.19479126	0.014996	0.014325	0.01771	0.004783	0.017614	0.077578	0.023986	0.024345	0.014309

Table S3. Exact p-values of the main analysis and all sensitivity analyses (continued)

	Main analysis		Sensitivity analyses										
Name	p-values unadjusted	p.values Holm adjusted	S5 p-values Holm adjusted	S6 p-values Holm adjusted	S7 p-values Holm adjusted	S8 p-values Holm adjusted	S9 p-values Holm adjusted	S10 p-values Holm adjusted	S11 p-values Holm adjusted	S12 p-values Holm adjusted	S13 p-values Holm adjusted	S14 p-values Holm adjusted	S15 p-values Holm adjusted
Onions	4.39891E-05	0.005323	0.114071849	0.183971356	0.001935	0.058017	0.002818	0.00616	0.01377	0.003702	0.004894	0.132166	0.005886
Orange juice	0.522735226	1	1	1	1	1	1	1	1	1	1	1	1
Oranges	0.171875776	1	1	1	1	1	1	1	1	1	1	1	1
Pasta	0.014864314	1	0.477241307	0.638496016	1	0.044581	1	1	1	0.955142	1	1	1
Pears	0.272595012	1	1	1	1	1	1	1	1	1	1	1	1
Pizza	0.002480187	0.250499	1	0.2240222	0.330511	0.008464	0.708729	1	0.216499	1	1	1	0.174724
Plain yogurt	0.000216095	0.024203	0.120230128	0.621754623	0.047231	0.630849	0.281882	0.166375	0.158023	0.078153	0.056976	0.015786	0.033883
Plums	0.143916376	1	1	1	1	1	1	1	1	1	1	1	1
Pollock	0.000863345	0.092378	1	1	0.143336	0.044581	1	0.470393	0.101583	1	1	0.29083	0.104673
Pork chop	0.157101817	1	1	1	1	1	1	1	1	1	1	1	1
Porridge	0.106132737	1	1	1	1	1	1	1	1	1	1	1	1
Potato crisps	0.117522128	1	1	1	1	1	1	1	1	1	1	1	1
Potatoes	0.74879117	1	1	1	1	1	1	1	1	1	1	1	1
Prawns	0.425532762	1	1	1	1	1	1	1	1	1	1	1	1
Raw carrots	0.003888101	0.384922	1	0.295768717	0.500444	0.370037	0.708729	0.246527	0.477047	1	1	1	0.470902
Red meat	0.236576004	1	1	1	1	1	1	1	1	1	1	1	1
Red wine	5.76256E-05	0.006915	0.005158746	0.039125901	0.025714	0.002988	0.129273	0.063654	0.052857	0.101384	0.225959	1	0.00611
Regular fizzy drinks	1.02536E-08	1.43E-06	7.07261E-06	0.00038049	5.36E-06	0.000331	2.21E-06	0.000818	1.78E-06	6.04E-06	5.79E-06	0.0005	2.29E-06
Roast chicken	0.997852569	1	1	1	1	1	1	1	1	1	1	1	1
Salad dressing	0.162435748	1	1	1	1	1	1	1	1	1	1	1	1
Salad leaves	1.3174E-05	0.001673	0.05822519	0.013030519	0.004516	0.002514	0.031379	0.003589	0.028751	0.101402	0.140418	0.063449	0.002303
Salami	0.746353045	1	1	1	1	1	1	1	1	1	1	1	1
Salmon	0.436274685	1	1	1	1	1	1	1	1	1	1	1	1
Salty foods	0.171968047	1	1	1	1	1	1	1	1	1	1	1	1
Salty pretzels	0.904653159	1	1	1	1	1	1	1	1	1	1	1	1
Sardines	0.462794343	1	1	1	1	1	1	1	1	1	1	1	1
Sausages (meat)	0.016330941	1	1	0.758179637	1	1	1	1	1	1	1	1	1
Savoury biscuits	0.448606208	1	1	1	1	1	1	1	1	1	1	1	1

Table S3. Exact p-values of the main analysis and all sensitivity analyses (continued)

	Main analysis		Sensitivity analyses										
Name	p-values unadjusted	p-values Holm adjusted	S5 p-values Holm adjusted	S6 p-values Holm adjusted	S7 p-values Holm adjusted	S8 p-values Holm adjusted	S9 p-values Holm adjusted	S10 p-values Holm adjusted	S11 p-values Holm adjusted	S12 p-values Holm adjusted	S13 p-values Holm adjusted	S14 p-values Holm adjusted	S15 p-values Holm adjusted
Shellfish	0.23424294	1	1	1	1	1	1	1	1	1	1	0.662176	1
Skimmed milk	0.003895254	0.384922	1	1	0.175063	0.062731	1	0.832607	0.124473	0.876403	0.145876	0.769051	0.432256
Smoked fish	0.082757008	1	1	1	1	1	1	1	1	1	1	1	1
Soft cheese	0.938578404	1	1	1	1	1	1	1	1	1	1	1	1
Soy sauce	0.197195988	1	1	1	1	1	1	1	1	1	1	1	1
Soya milk	0.339409077	1	1	1	1	1	1	1	1	1	1	1	1
Spicy foods	3.05714E-05	0.003791	0.04625246	0.025195366	0.002006	0.047248	0.002305	0.00271	0.009565	0.042325	0.005767	0.050293	0.003806
Spinach	7.46136E-07	1E-04	0.072661373	0.006665954	0.000894	0.008372	0.007645	0.000335	0.005778	0.005822	0.000376	0.029705	0.00016
Spirits	4.08582E-06	0.000535	0.021620613	0.003117833	0.000113	0.013546	0.001446	0.000367	0.000982	0.001821	0.000201	0.065983	0.000431
Strawberries	0.16211378	1	1	1	1	1	1	1	1	1	1	1	1
Sweet coffee house drinks	0.0064531	0.613044	1	0.624592387	0.986691	1	0.126107	0.480944	0.472418	1	0.516599	0.021826	0.712074
Sweet foods	0.486099859	1	1	1	1	1	1	1	1	1	1	1	1
Tea with sugar	6.20668E-05	0.007386	0.046337974	0.00037917	0.001975	0.033729	0.000217	0.002425	0.002791	0.01136	0.02405	0.224879	0.008012
Tea without sugar	0.005687887	0.546037	0.533755467	0.406367951	0.743598	1	0.447691	0.988417	0.86346	1	1	0.662176	0.580573
Tomato ketchup	0.425132081	1	1	1	1	1	1	1	1	1	1	1	1
Tinned tuna	7.38048E-06	0.000959	0.002432212	0.042312261	0.000718	0.035324	0.050204	0.017696	9.36E-05	0.001349	0.143194	0.000862	0.001009
Turnip (white)	0.108026848	1	1	1	1	1	1	1	1	1	1	1	1
Vegetables	0.008320211	0.757139	1	1	1	1	1	1	1	1	0.811666	0.429551	0.89591
Vinegar	0.095184744	1	1	1	1	1	1	1	1	1	1	1	1
Whisky	0.04728473	1	1	1	1	1	1	1	1	1	1	1	1
White bread	0.001741738	0.177657	0.385106028	0.214176986	0.805252	0.046233	0.475964	1	1	0.216311	0.497338	1	0.279347
White rice	0.840322043	1	1	1	1	1	1	1	1	1	1	1	1
White wine	6.69969E-05	0.007906	0.003020769	0.004218097	0.027858	0.030781	0.099074	0.113999	0.026096	0.037157	0.103059	0.848217	0.006068
Wholegrain breakfast cereal	0.021197888	1	1	1	1	0.53301	1	1	1	1	1	1	1
Whole milk	8.88738E-05	0.010398	0.004162348	0.20327436	0.010518	0.349487	0.023113	0.028068	0.026096	0.089538	0.002279	0.557768	0.009494
Wholemeal bread	2.88557E-06	0.000381	0.037962812	0.041630475	0.000646	5.22E-06	0.004393	0.001777	0.003596	0.021545	0.006109	0.409446	0.000408

Table S3. Exact p-values of the main analysis and all sensitivity analyses (continued)

Parameters	High preference for alcoholic beverages (n = 58,445)	High preference for cheese (n = 114,275)	High preference for fish (n = 127,034)	High preference for fruit (n = 151,884)	High preference for meat (n = 122,047)	High preference for sweets (n = 100,303)	High preference for vegetables (n = 121,078)
Age at completion of FPQ	66 (8)	66 (8)	66 (8)	66 (8)	66 (8)	66 (8)	66 (8)
BMI (kg/m^2)							
- Underweight (< 18.5 kg/m^2)	168 (0.3)	582 (0.5)	608 (0.5)	851 (0.6)	435 (0.4)	442 (0.4)	671 (0,6)
- Normal (18.5 – 25 kg/m^2)	20,004 (34.2)	43,760 (38.3)	48,626 (38.3)	60,061 (39.5)	41,524 (34.0)	37,505 (37.4)	49,074 (40.5)
- Overweight (25 – 30 kg/m^2)	26,946 (46.1)	47,761 (41.8)	53,583 (42.2)	62,344 (41.0)	53,409 (43.8)	42,422 (42.3)	49,598 (41.0)
- Obese (>30 kg/m^2)	11,327 (19.4)	22,172 (19.4)	24,217 (19.1)	28,628 (18.8)	26,679 (21.9)	19,934 (19.9)	21,735 (18.0)
Sex – female	19,278 (33.0)	62,783 (54.9)	70,675 (55.6)	90,266 (59.4)	62,931 (51.6)	55,511 (55.3)	72,138 (59.6)
Ethnic background							
- White	57,380 (98.2)	112,033 (98.0)	123,790 (97.4)	147,568 (97.2)	119,739 (98.1)	98,199 (97.9)	117,614 (97.1)
- Mixed, Asian, Black, Chinese, and other	1,065 (1.8)	2,242 (2.0)	3,244 (2.6)	4,316 (2.8)	2,308 (1.9)	2,104 (2.1)	3,464 (2.9)
General health status							
- Poor	1,051 (1.8)	2,269 (2.0)	2,432 (1.9)	3,064 (2.0)	2,803 (2.3)	2,177 (2.2)	2,172 (1.8)
- Fair	8,460 (14.4)	16,285 (14.3)	18,367 (14.5)	22,096 (14.5)	19,516 (16.0)	15,241 (15.2)	16,446 (13.6)
- Good	35,339 (60.5)	69,092 (60.5)	77,217 (60.8)	92,118 (60.7)	74,079 (60.7)	60,925 (60.7)	73,499 (60.7)
- Excellent	13,595 (23.3)	26,629 (23.3)	29,018 (22.8)	34,606 (22.8)	25,649 (21.0)	21,960 (21.9)	28,961 (23.9)
Highest qualification							
- None of the below	3,077 (5.3)	6,740 (5.9)	8,448 (6.7)	10,114 (6.7)	9,022 (7.4)	6,692 (6.7)	6,531 (5.4)
- National exams at age 16 years	6,449 (11.0)	14,705 (12.9)	17,436 (13.7)	21,186 (13.9)	18,308 (15.0)	14,500 (14.5)	15,452 (12.8)
- Vocational qualifications or optional national exams at ages 17-18 years	10,231 (17.5)	18,936 (16.6)	21,899 (17.2)	25,966 (17.1)	22,698 (18.6)	17,895 (17.8)	19,979 (16.5)
- Professional	8,635 (14.8)	17,900 (15.7)	20,371 (16.0)	24,260 (16.0)	19,901 (16.3)	16,161 (16.1)	19,131 (15.8)
- College or University	30,053 (51.4)	55,994 (49.0)	58,880 (46.3)	70,358 (46.3)	52,118 (42.7)	45,055 (44.9)	59,985 (49.5)
Smoking status							
- Never	30,009 (51.3)	64,164 (56.1)	72,429 (57.0)	89,397 (58.9)	70,028 (57.4)	60,471 (60.3)	68,706 (56.7)
- Previous	24,293 (41.6)	42,513 (37.2)	46,340 (36.5)	53,152 (35.0)	43,502 (35.6)	33,816 (33.7)	44,540 (36.8)
- Occasional	2,019 (3.5)	3,041 (2.7)	3,201 (2.5)	3,516 (2.3)	2,999 (2.5)	2,260 (2.3)	3,153 (2.6)
- Current <10 cigarettes per day	645 (1.1)	1,325 (1.2)	1,441 (1.1)	1,713 (1.1)	1,383 (1.1)	1,057 (1.1)	1,443 (1.2)
- Current 10 to 14 cigarettes per day	499 (0.9)	1,061 (0.9)	1,205 (0.9)	1,388 (0.9)	1,292 (1.1)	875 (0.9)	1,119 (0.9)
- Current 15 to 19 cigarettes per day	379 (0.6)	852 (0.7)	969 (0.8)	1,106 (0.7)	1,103 (0.9)	757 (0.8)	888 (0.7)
- Current ≥20 cigarettes per day	601 (1.0)	1,319 (1.2)	1,449 (1.1)	1,612 (1.1)	1,740 (1.4)	1,067 (1.1)	1,229 (1.0)

Table S4. Baseline characteristics after food preference group (high preference: 7-9)

Parameters	Low preference for alcoholic beverages (n = 26,596)	Low preference for cheese (n = 8,389)	Low preference for fish (n = 7,371)	Low preference for fruit (n = 377)	Low preference for meat (n = 11,078)	Low preference for sweets (n = 2,460)	Low preference for vegetables (n = 488)
Age at completion of FPQ	67 (8)	65 (8)	66 (8)	66 (8)	64 (8)	66 (8)	64 (8)
BMI (kg/m^2)							
- Underweight (< 18.5 kg/m^2)	239 (0.9)	72 (0.9)	73 (1.0)	1 (0.3)	173 (1.6)	47 (1.9)	1 (0.2)
- Normal (18.5 – 25 kg/m^2)	10,517 (39.5)	3,341 (39.8)	3,075 (41.7)	118 (31.3)	6213 (56.1)	1,036 (42.1)	132 (27.0)
- Overweight (25 – 30 kg/m^2)	9,780 (36.8)	3,305 (39.4)	2,682 (36.4)	148 (39.3)	3464 (31.3)	912 (37.1)	209 (42.8)
- Obese (>30 kg/m^2)	6,060 (22.8)	1,671 (19.9)	1,541 (20.9)	110 (29.2)	1228 (11.1)	465 (18.9)	146 (29.9)
Sex – female	20,715 (77.9)	4,816 (57.4)	4,667 (63.3)	159 (42.2)	8,142 (73.5)	1,484 (60.3)	171 (35.0)
Ethnic background							
- White	25,522 (96.0)	7,928 (94.5)	7,169 (97.3)	363 (96.3)	10,637 (96.0)	2,317 (94.2)	480 (98.4)
- Mixed, Asian, Black, Chinese, and other	1,074 (4.0)	461 (5.5)	202 (2.7)	14 (3.7)	441 (4.0)	143 (5.8)	8 (1.6)
General health status							
- Poor	1,000 (3.8)	290 (3.5)	292 (4.0)	22 (5.8)	265 (2.4)	67 (2.7)	30 (6.1)
- Fair	5,099 (19.2)	1,494 (17.8)	1,298 (17.6)	102 (27.1)	1,405 (12.7)	414 (16.8)	130 (26.6)
- Good	15,550 (58.5)	5,009 (59.7)	4,201 (57.0)	198 (52.5)	6,426 (58.0)	1,366 (55.5)	264 (54.1)
- Excellent	4,947 (18.6)	1,596 (19.0)	1,580 (21.4)	55 (14.6)	2,982 (26.9)	613 (24.9)	64 (13.1)
Highest qualification							
- None of the below	2,956 (11.1)	833 (9.9)	500 (6.8)	48 (12.7)	424 (3.8)	210 (8.5)	66 (13.5)
- National exams at age 16 years	4,833 (18.2)	1,474 (17.6)	1,030 (14.0)	75 (19.9)	1,138 (10.3)	317 (12.9)	106 (21.7)
- Vocational qualifications or optional national exams at ages 17-18 years	4,700 (17.7)	1,633 (19.5)	1,348 (18.3)	85 (22.5)	1,544 (13.9)	424 (17.2)	93 (19.1)
- Professional	4,599 (17.3)	1,368 (16.3)	1,080 (14.7)	59 (15.6)	1,532 (13.8)	357 (14.5)	96 (19.7)
- College or University	9508 (35.7)	3,081 (36.7)	3,413 (46.3)	110 (29.2)	6,440 (58.1)	1,152 (46.8)	127 (26.0)
Smoking status							
- Never	17,417 (65.5)	5,360 (63.9)	4,296 (58.3)	189 (50.1)	6,317 (57.0)	1,212 (49.3)	304 (62.3)
- Previous	7,350 (27.6)	2,492 (29.7)	2,477 (33.6)	123 (32.6)	4,088 (36.9)	1,019 (41.4)	130 (26.6)
- Occasional	319 (1.2)	135 (1.6)	175 (2.4)	13 (3.4)	287 (2.6)	67 (2.7)	12 (2.5)
- Current <10 cigarettes per day	330 (1.2)	93 (1.1)	107 (1.5)	10 (2.7)	146 (1.3)	42 (1.7)	7 (1.4)
- Current 10 to 14 cigarettes per day	361 (1.4)	78 (0.9)	93 (1.3)	13 (3.4)	92 (0.8)	36 (1.5)	8 (1.6)
- Current 15 to 19 cigarettes per day	323 (1.2)	84 (1.0)	80 (1.1)	12 (3.2)	59 (0.5)	33 (1.3)	13 (2.7)
- Current ≥20 cigarettes per day	496 (1.9)	147 (1.8)	143 (1.9)	17 (4.5)	89 (0.8)	51 (2.1)	14 (2.9)

Table S5. Baseline characteristics after food preference group (low preference: 1-3)

Table S6. Baseline Characteristics by FPQ Completion*

Parameters	FPQ not completed (n = 317,514)	FPQ completed (n = 181,051)
**Age, years**	57 (8)	56 (8)
**Sex**		
- Female	167,929 (52.9)	103,299 (57.1)
- Male	149,585 (47.1)	77,752 (42.9)
**BMI (kg/m^2)**		
- Underweight (< 18.5 kg/m^2)	1,578 (0.5)	1,012 (0.6)
- Normal (18.5 – 25 kg/m^2)	91,614 (29.1)	69,481 (38.5)
- Overweight (25 – 30 kg/m^2)	135,889 (43.2)	74,651 (41.3)
- Obese (>30 kg/m^2)	85,818 (27.3)	35,475 (19.6)
- Missing	2,615 (0.8)	432 (0.2)
**Ethnic background**		
- White	293,782 (93.1)	175,193 (97.1)
- Mixed, Asian, Black, Chinese, and other	21,636 (6.9)	5,214 (2.9)
- Missing	2,096 (0.7)	644 (0.4)
**General health status**		
- Poor	18,329 (5.8)	4,219 (2.3)
- Fair	76,249 (24.2)	28,260 (15.6)
- Good	177,878 (56.6)	108,980 (60.3)
- Excellent	42,039 (13.4)	39,174 (21.7)
- Missing	3,019 (1.0)	418 (0.2)
**Highest qualification**		
- None of the below	72,373 (23.2)	12,881 (7.1)
- National exams at age 16 years	57,461 (18.4)	25,812 (14.3)
- Vocational qualifications or optional national exams at ages 17-18 years	58,659 (18.8)	31,532 (17.5)
- Professional	43,742 (14.0)	28,667 (15.9)
- College or University	79,650 (25.5)	81,450 (45.2)
- Missing	5,629 (1.8)	709 (0.4)
**Smoking status**		
- Never	166,850 (53.3)	104,638 (58.2)
- Previous	108,616 (34.7)	63,121 (35.1)
- Occasional	9,299 (3.0)	4,301 (2.4)
- Current <10 cigarettes per day	5,046 (1.6)	2,111 (1.2)
- Current 10 to 14 cigarettes per day	6,117 (2.0)	1,809 (1.0)

- Current 15 to 19 cigarettes per day	5,857 (1.9)	1,501 (0.8)
- Current ≥20 cigarettes per day	11,018 (3.5)	2,325 (1.3)
- Missing	4,711 (1.5)	1,245 (0.7)

* Categorical variables are summarised as frequencies (percentages) and continuous variables as mean (standard deviation). Abbreviations: BMI, Body Mass Index; FPQ, Food Preference Questionnaire.
